# Lanthanide and Actinide‐Centered Polyoxo‐Noble‐Metalate‐Based Metal−Organic Frameworks

**DOI:** 10.1002/asia.202500737

**Published:** 2025-07-08

**Authors:** Saurav Bhattacharya, Anupam Sarkar, Tsedenia A. Zewdie, Alina J. George, Samer Dawoud, Talha Nisar, Christian J. Schürmann, Veit Wagner, Laurent Ruhlmann, Ulrich Kortz

**Affiliations:** ^1^ School of Science Constructor University Campus Ring 1 Bremen 28759 Germany; ^2^ Department of Chemistry Birla Institute of Technology and Science, Pilani K K Birla Goa Campus Zuarinagar Sancoale Goa 403726 India; ^3^ Institute of Chemistry (UMR au CNRS n°7177) University of Strasbourg 4 rue Blaise Pascal Strasbourg France; ^4^ Rigaku Europe SE Hugenottenallee 167 Neu‐Isenburg 63263 Germany

## Abstract

This paper reports the synthesis and the characterization of the first Ce^III^‐centered polyoxopalladate‐based metal‐organic framework (POP‐MOF), **Ce‐JUB‐1**, and the first actinide‐centered POP‐MOF, **Th‐JUB‐1**, along with lanthanide‐centered POP‐MOFs, **Ln‐JUB‐1** (Ln^III^ = Pr, Nd, Sm, Eu, Gd, Tb, Dy, Ho, Er, Tm, Yb, Lu). The compounds have been characterized by various solid‐state techniques, such as single‐crystal and powder X‐ray diffraction, IR spectroscopy, and XPS spectroscopy. In addition, the oxidation state of the central cerium ion in **Ce‐JUB‐1** as well as the nature of its redox behavior have been ascertained using detailed electrochemical studies, which exhibit a quasi‐reversible oxidation process without indicating any degradation of the compound. The discovery of an actinide‐centered POP‐MOF, **Th‐JUB‐1**, is important as it opens up avenues for the use of POPs as radiopharmaceutical agents.

## Graphical abstract

The synthesis and the structural characterization of the first Ce^III^‐centered polyoxopalladate‐based metal‐organic framework (POP‐MOF), **Ce‐JUB‐1**, and the first actinide‐centered POP‐MOF, **Th‐JUB‐1**, along with lanthanide‐centered POP‐MOFs, **Ln‐JUB‐1** have been reported. In addition, the redox behavior of the central Ce^III^ ion in **Ce‐JUB‐1** has been ascertained using detailed electrochemical studies.

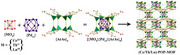

## 1 Introduction

Polyoxo‐noble‐metalates are variants of the class of materials known as polyoxometalates (POMs) with the addenda atoms comprising noble metals.^[^ ^1^ ^]^ These materials seamlessly integrate the best features of both POMs as well as noble metals, viz., the structural integrity and tunability of POMs and the catalytic behavior of the noble metal sites. Polyoxopalladates (POPs), which are polyoxo‐noble‐metalates with Pd as the addenda atom, constitute the largest subgroup, with over 80 molecules of diverse compositions and shapes isolated and characterized.^[^ ^1^ ^]^ Similar to the oft‐observed Keggin and Wells‐Dawson structural topologies in POMs, the POPs have been shown to possess their own structural topologies, viz. the nano‐cube [Pd_12_O_8_L_8_]^x−^ and the nanostar [Pd_15_O_10_L_10_]^x−^, L being the capping heterogroups, such as phosphate, phenylphosphonate, arsenate, phenylarsonate, and dimethylarsinate. These cage‐like structures have been shown to act as hosts that can encapsulate a wide variety of guest metal ions (∼40 elements from the periodic table, including transition metals, main group elements, and lanthanides), leading to the M‐centered POPs [MPd_12_O_8_L_8_]^x−^ and [MPd_15_O_10_L_10_]^x−^ (M = central hetero‐metal‐ion), the structural type adopted being dependent on the ionic radii and charges of M as well as on the heterogroup used.^[^ ^2^ ^]^ The synergistic effects between the various M ions and the POP cages as a consequence of the coordination micro‐confinement have enabled researchers to utilize these materials as bimetallic heterogeneous catalysts,^[^ ^3^ ^]^ radiopharmaceuticals,^[^ ^4^ ^]^ as well as molecular magnetic materials, especially in the area of molecular spin qubits.^[^ ^5^ ^]^ For example, the lanthanide‐centered POP nanocubes, [Ln^III^Pd_12_O_8_(PhAs)_8_]^5−^ (Ln^III^ = Dy, Ho, Er, Tb, and Tm) have exhibited molecular spin qubit behavior.

Through the judicious use of synthetic strategies, we have shown before that the nano‐cubic arsenate‐capped POP and [Pd_13_O_8_(AsO_4_)_8_]^14−^ can be utilized as a secondary building unit (SBU) and confined as the component of a three‐dimensional metal‐organic framework (MOF), where the organic component is a suitable arylarsonate.^[^ ^6^ ^]^ This led to the successful isolation of the first POP‐MOF, **JUB‐1**, with the formula [Pd_13_Ba_8_O_8_(CPA)_8_](NO_3_)_2_·(guest molecules), where CPA = ‐carboxyphenylarsonate. **JUB‐1** exhibited good heterogeneous catalytic activity in microwave‐assisted Suzuki–Miyaura C─C coupling reactions. The primary motivation of this work was the possibility of isolating Ce^III^ and other lanthanides as well as actinide‐centered POP‐based MOFs (Ac‐POP‐MOFs), which have been hitherto elusive in the synthesis of discrete molecular polyoxopalladates. Actinides are gaining ground as potential radiopharmaceutical agents for anticancer therapy^[^ ^7^ ^]^ and, therefore, the incorporation of actinides in the nano‐cubic POP would lead to novel therapeutic materials. Similar studies on ^224^Ra and ^205/206^Bi‐labeled POPs have shown promising results before.^[^ ^4^ ^]^ Here we report on a systematic extension of the above‐mentioned work.

## 2 Experimental Section

### 2.1 Materials

All chemicals were purchased commercially and utilized without further purification. For the synthesis of 4‐carboxyphenylarsonic acid (CPAH_3_), we followed the same modified procedure as published by us in 2019.^[^ ^6^ ^]^ The sodium dimethylarsinate buffer solution can be prepared either by titrating an aqueous solution of dimethylarsinic acid with NaOH or by titrating an aqueous solution of sodium dimethylarsinate with acetic acid until the pH of the resulting solution reaches 7. However, the buffer synthesized utilizing the former method was used exclusively for the syntheses below.

### 2.2 Synthesis of Na[CePd_12_Ba_6_O_8_(CPA)_8_]·10NaNO_3_·8[(CH_3_)_2_AsO_2_H]·50H_2_O, **Ce‐JUB‐1**

Compound **Ce‐JUB‐1** was synthesized by suspending palladium(II) acetate, Pd(OAc)_2_ (22.4 mg, 0.1 mmol), 4‐carboxyphenylarsonic acid, H_2_O_3_AsC_6_H_4_‐4‐COOH (CPAH_3_) (24.6 mg, 0.1 mmol), and cerium(III) nitrate, Ce(NO_3_)_3_⋅6H_2_O (21.7 mg, 0.05 mmol) in 2 mL of sodium dimethylarsinate (trivial name: sodium cacodylate) buffer solution (0.5 M, pH 7) and heating at 70 °C whilst stirring for an hour. Over the course of heating, the heterogeneous mixture turned into a red solution with a dark‐red precipitate. After 1 h, the reaction mixture was cooled, and the pH was adjusted to 7.0 utilizing a 6 M aqueous NaOH solution (pH after heating ∼5.4). The solution was then heated further at 70 °C for 1.5 h. Subsequently, the solution was cooled, centrifuged for 30 min (4000 rpm), and filtered. The deep red filtrate was then layered with 150 µL of 0.5 M aq. Ba(NO_3_)_2_ solution and kept in an open vial for crystallization. Dark‐red rod‐shaped crystals were obtained after 10 days, which were filtered and dried in air (∼28% yield based on Pd). Elemental analysis (%) calculated for Na[CePd_12_Ba_6_O_8_(C_7_H_4_AsO_5_)_8_]·10 NaNO_3_·8[(CH_3_)_2_AsO_2_H]·50 H_2_O, (**Ce‐JUB‐1**): C 12.02, H 2.63, N 1.9, Pd 17.7, Na 3.52, As 16.7, Ce 2.00, Ba 11.45; found: C 12.04, H 2.38, N 1.11, Pd 17.2, Na 3.53, As 17.1, Ce 2.42, Ba 11.4.

### 2.3 Synthesis of [ThPd_12_Ba_6_O_8_(CPA)_8_]·Ba(NO_3_)_2_·8NaNO3·10[(CH_3_)_2_AsO_2_H]·50H_2_O, **Th‐JUB‐1**

Compound **Th‐JUB‐1** was synthesized by suspending palladium(II) acetate, Pd(OAc)_2_ (22.4 mg, 0.1 mmol), 4‐carboxyphenylarsonic acid, H_2_O_3_AsC_6_H_4_‐4‐COOH (CPAH_3_) (24.6 mg, 0.1 mmol), and thorium(IV) nitrate, Th(NO_3_)_4_⋅4H_2_O (27.6 mg, 0.05 mmol) in 2 mL of sodium dimethylarsinate (trivial name: sodium cacodylate) buffer solution (0.5 M, pH 7) and heating at 70 °C whilst stirring for an h. Over the course of heating, the heterogeneous mixture turned into a red solution with a dark–red precipitate. After 1 h, the reaction mixture was cooled, and pH was adjusted to 7.0 utilizing a 6 M aqueous NaOH solution (pH after heating ∼5.3). The solution was then heated further at 70 °C for 1.5 h. Subsequently, the solution was cooled, centrifuged for 30 min (4000 rpm), filtered. The deep red filtrate was then layered with 150 µL of 0.5 M aq. Ba(NO_3_)_2_ solution and kept in an open vial for crystallization. Dark‐red rod‐shaped crystals were obtained after 10 days, which were filtered and dried in air (∼36% yield based on Pd). Elemental analysis (%) calculated for [ThPd_12_Ba_6_O_8_(C_7_H_4_AsO_5_)_8_]·Ba(NO_3_)_2_·8 NaNO_3_·10[(CH_3_)_2_AsO_2_H]·50 H_2_O, (**Th‐JUB‐1**): C 11.97, H 2.67, N 1.84, Pd 16.74, Na 2.41, As 17.70, Th 3.10, Ba 12.60; found: C 12.31, H 2.57, N 1.32, Pd 16.30, Na 2.55, As 18.4, Th 3.70, Ba 12.20.

### 2.4 Synthesis of the Other **Ln‐JUB‐1**

The other **Ln‐JUB‐1** compounds were synthesized in a manner analogous to the synthesis of **Ce‐JUB‐1** albeit changing the lanthanide salts (PrCl_3_⋅6H_2_O for **Pr‐JUB‐1**, Nd(CH_3_COO)_3_⋅H_2_O for **Nd‐JUB‐1**, Sm(NO_3_)_3_⋅6H_2_O for **Sm‐JUB‐1**, EuCl_3_·6H_2_O for **Eu‐JUB‐1**, GdCl_3_·6H_2_O for **Gd‐JUB‐1**, TbCl_3_·6H_2_O for **Tb‐JUB‐1**, DyCl_3_·6H_2_O for **Dy‐JUB‐1**, HoCl_3_·6H_2_O for **Ho‐JUB‐1**, ErCl_3_·6H_2_O for **Er‐JUB‐1**, TmCl_3_·H_2_O for **Tm‐JUB‐1**, YbCl_3_·6H_2_O for **Yb‐JUB‐1**, and Lu(NO_3_)_3_ for **Lu‐JUB‐1**. The elemental analysis of two of the Ln‐JUB‐1, namely **Eu‐JUB‐1** and **Gd‐JUB‐1** as representatives, was performed using ICP‐MS and ICP‐OES techniques. For the **Eu‐JUB‐1**, the elemental composition (in g/kg) was found to be 23 for Eu, 199 for Pd, 179 for As, 138 for Ba, and 36.5 for Na, which translates to an elemental ratio Eu:Pd:As:Ba:Na of 1:12.3:15.8:6.6:10.5. Similarly, for the **Gd‐JUB‐1**, the elemental composition (in g/kg) was found to be 25 for Gd, 194 for Pd, 187 for As, 136 for Ba, and 37 for Na, which translates to an elemental ratio Gd:Pd:As:Ba:Na of 1:11.5:15.7:6.2:10.2. Both these results match fairly well with the formula derived for the **Ce‐JUB‐1**, indicating that the compositions must be analogous.

## 3 Results and Discussion

We have successfully synthesized and structurally characterized the first Ce^III^‐centered, and the first actinide‐centered POP‐MOFs, viz. Na[Ce^III^Pd_12_Ba_6_O_8_(CPA)_8_]·10NaNO_3_·8[(CH_3_)_2_AsO_2_H]·50H_2_O (**Ce‐JUB‐1**) and [Th^IV^Pd_12_Ba_6_O_8_(CPA)_8_]·Ba(NO_3_)_2_·8NaNO_3_·10[(CH_3_)_2_AsO_2_H]·50H_2_O (**Th‐JUB‐1**) along with several other lanthanide‐centered POP‐MOFs (**Ln‐JUB‐1**, Ln^III^ = Pr, Nd, Sm, Eu, Gd, Tb, Dy, Ho, Er, Tm, Yb, Lu). 4‐carboxyphenylarsonic acid was synthesized using a previously reported procedure.^[^ ^6^ ^]^ **Ce‐JUB‐1**, **Th‐JUB‐1**, and the **Ln‐JUB‐1** were synthesized using analogous procedures wherein a mixture of CPAH_3_, Pd(OAc)_2_, and Ce(NO_3_)_3_·6H_2_O for **Ce‐JUB‐1** or Th(NO_3_)_4_·4H_2_O for **Th‐JUB‐1** or other lanthanide salts for the **Ln‐JUB‐1** in a sodium dimethylarsinate (commonly referred to as “cacodylate”) buffer solution (0.5 M, pH 7) were heated at 70 °C, maintaining the pH at ∼7.0 in‐between with aq. NaOH, and subsequently layering the resulting solution with aq. Ba(NO_3_)_2_ solution (see experimental section above for further details). The resulting dark red single crystals were filtered, washed with acetonitrile, air‐dried, and subsequently utilized for further characterizations. The use of the sodium cacodylate buffer solution was deemed necessary for the syntheses, as reactions in the absence of cacodylate led to very poor yields. A point to note that Ce^III^ salts, viz., Ce(NO_3_)_3_ or CeCl_3_ as reactants were found to yield **Ce‐JUB‐1**, whereas repeated attempts with Ce^IV^ salts, such as Ce(SO_4_)_2_. (NH_4_)_2_Ce(NO_3_)_6_, or CeO_2_ did not yield the desired **Ce‐JUB‐1**. Interestingly, the Ce^IV^‐centered discrete polyoxo‐12‐palladate cube [Ce^IV^Pd_12_O_8_(AsO_4_)_8_]^12−^ was recently reported,^[^ ^2b^ ^]^ wherein the cluster was synthesized by reaction of Pd(OAc)_2_, Ce^IV^(NO_3_)_4_, Na_2_HAsO_4_ and KVO_3_ in a NaOAc buffer solution. These authors observed that their reaction condition prevented the incorporation of a Ce^III^ guest ion despite employing a Ce^III^ precursor salt, probably due to the oxidation of Ce^III^ to Ce^IV^ during the reaction (KVO_3_ is an oxidizing agent).

**Figure 1:**
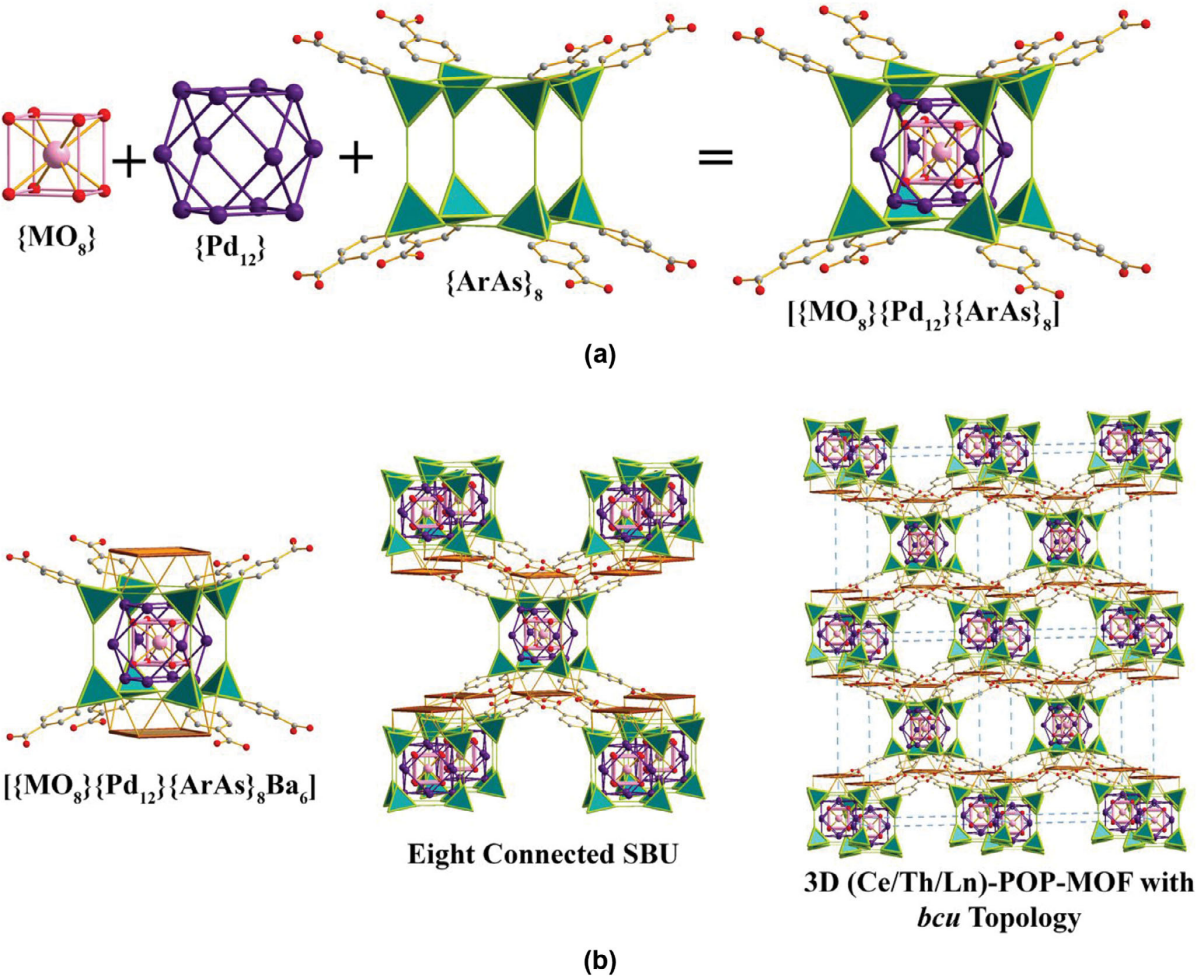
a) The onion‐like layered assembly of the POP SBU comprising the {MO_8_} cubic entity, the cuboctahedral {Pd_12_} moiety, and the {ArAs}_8_ cubic unit. (Ar = 4‐carboxyphenyl; color code: M = pink balls, Pd = purple balls, arsonate = green polyhedral, O = red balls, and C = grey balls). b) The SBU [{MO_8_}{Pd_12_}{ArAs}_8_Ba_6_] connected to eight other SBUs leading to the formation of the 3D (Ce/Th/Ln)‐POP‐MOF with bcu topology.

Single crystal X‐ray diffraction (SC‐XRD) experiments revealed that both the **Ce‐JUB‐1** and the **Th‐JUB‐1** crystallize in the tetragonal *P*4/*mnc* space group and possess similar unit cell parameters (Table 1). The asymmetric units of both the compounds are made up of one crystallographically distinct Ce^3+^ or Th^4+^, (site occupancy of 0.125), respectively, two Pd^2+^ ions (site occupancies of 0.5 and 1, respectively), one fully occupied CPA^3−^ linker, one Ba^2+^ ion with a site occupancy of 0.75 and one μ_4_‐O atom, which lead to the overall framework formulae of [(Ce^III^)Pd_12_Ba_6_(μ_4_‐O)_8_(CPA)_8_]^−^ and [(Th^IV^)Pd_12_Ba_6_(μ_4_‐O)_8_(CPA)_8_] (Figure S1), the negative charge in the former being balanced by extra‐framework Na^+^ ion as evidenced from elemental analysis (see experimental section above). The oxidation states on the Ce and the Th were obtained via XPS analysis (vide infra). In terms of the structural arrangement, the {MO_8_} cubic entity, where M = Ce^3+^ or Th^4+^, is encapsulated by the cuboctahedral {Pd_12_} moiety, which is further encased by the {ArAs}_8_ cubic unit (Ar = 4‐carboxyphenyl unit), leading to a multi‐layered onion‐like assembly (Figure 1a). The M─O bond distances in the {MO_8_} cubic entity were found to be 2.348(3) Å for M = Ce^3+^ and 2.402(10) Å for M = Th^4+^ (Table S1). The Pd^2+^ ions in the cuboctahedral {Pd_12_} moiety exhibit the square planar geometry with the coordination environment formed out of the connectivity with two oxygens of the arsonate group of the CPA^3−^ linker and with two μ_4_‐O^2−^ (Pd─O bond distances in the range of 1.993(3)–2.034(3) Å for **Ce‐JUB‐1** and 1.979(12)–2.037(12) Å for **Th‐JUB‐1**). One pair of opposite sides of the [{MO_8_}{Pd_12_}{ArAs}_8_] nano‐cubic unit is connected to three Ba^2+^ ions each via coordination with the arsonate group oxygens (Figure 1b). Although the Ba^2+^ ions appear to be present as a tetranuclear oxo‐cluster decorating the opposite sides (the Ba^2+^ ions being connected to each other via μ_2_‐H_2_O and μ_2_‐O groups of the carboxylates), the site occupancy of Ba^2+^ ions being 0.75 results in a positional disorder wherein six Ba^2+^ ions occupy eight crystallographic sites around the nano‐cube. This is corroborated by elemental analysis studies (see Experimental Section above). The presence of six Ba^2+^ ions per formula unit as compared to eight Ba^2+^ ions per formula unit observed in the Pd‐centered **JUB‐1** ^6^ can be attributed to the higher charged central metal ion M that reduces the need for additional cations to ensure charge neutrality. Thus, the composite units {MPd_12_As_8_Ba_6_O_48_} (M = Ce^3+^ for **Ce‐JUB‐1** and Th^4+^ for **Th‐JUB‐1**) act as eight‐connected SBUs and are connected to other such units via the CPA^3−^ organic linkers leading to the formation of a three‐dimensional framework with a body‐centered cubic topology (bcu) possessing cylindrical channels along the crystallographic “*a*” direction (Figure 1b) harboring the guest molecules. A point to note is that although the other **Ln‐JUB‐1** compounds crystallize with different unit cell parameters (orthorhombic *Pnma* space group), they exhibit a framework structure analogous to the **Ce/Th‐JUB‐1**. The only difference is that the lower symmetry space group in **Ln‐JUB‐1** as compared to the **Ce/Th‐JUB‐1** removes the symmetry restrictions and, therefore, the positional disorder on the Ba^2+^ ions (Tables 1 and 2). Due to this reason, the [{LnO_8_}{Pd}_12_{ArAs}_8_}] nano‐cubic unit is decorated by the fully occupied trinuclear Ba_3_‐oxo cluster (bridged by nitrate anions) on two opposite sides (Figure S2).

**Table 1:** Single crystal data and structure refinement parameters for the compounds (CCDC No. 2443915–2443921).

Compound	Ce‐JUB‐1	Th‐JUB‐1	Pr‐JUB‐1	Nd‐JUB‐1	Sm‐JUB‐1	Eu‐JUB‐1	Gd‐JUB‐1
Empirical formula	CePd_12_As_8_Ba_6_C_56_H_32_O_48_	ThPd_12_As_8_Ba_6_C_56_H_32_O_48_	PrPd_12_As_8_Ba_6_C_56_H_32_O_68_N_2_	NdPd_12_As_8_Ba_6_C_56_H_32_O_66_N_2_	SmPd_12_As_8_Ba_6_C_56_H_32_O_68_N_2_	EuPd_12_As_8_Ba_6_C_56_H_32_O_70_N_2_	GdPd_12_As_8_Ba_6_C_56_H_32_O_68_N_2_
Formula weight (g/mol)	4313.13 (7190.99)*	5091.75 (7627.27)*	4661.94	4633.27	4671.38	4704.99	4678.28
Crystal system	Tetragonal	Tetragonal	Orthorhombic	Orthorhombic	Orthorhombic	Orthorhombic	Orthorhombic
Space group	*P*4*/mnc*	*P*4*/mnc*	*Pnma*	*Pnma*	*Pnma*	*Pnma*	*Pnma*
*a* (Å)	20.0424(2)	20.0273(5)	30.0770(5)	30.0776(8)	29.7241(6)	29.7760(11)	29.8428(6)
*b* (Å)	20.0424(2)	20.0273(5)	26.6499(4)	26.8157(7)	27.6017(6)	27.3771(11)	27.2647(6)
*c* (Å)	25.5360(3)	25.8970(8)	28.1155(5)	28.0210(8)	27.5604(5)	27.7084(13)	27.8408(7)
α (⁰)	90	90	90	90	90	90	90
ß (⁰)	90	90	90	90	90	90	90
γ (⁰)	90	90	90	90	90	90	90
Volume (Å^3^)	10257.8(2)	10387.1(6)	22536.0(6)	22600.4(11)	22611.5(8)	22587.3(16)	22652.8(9)
Z	2	2	4	4	4	4	4
D_calc._ (gm/cm^3^)	1.396	1.408	1.374	1.362	1.372	1.384	1.372
Absorption Coefficient (mm^−1^)	3.691	4.144	3.386	3.390	3.419	3.442	3.447
F(000)	3924	3988	8548	8488	8560	8628	8568
θ Range for data collection (deg)	2.272–26.368	1.285–24.999	2.401–25.999	1.537–25.027	1.556–25.026	1.553–25.027	1.548–25.027
Completeness to θ*_max_* (%)	99.7	99.8	99.8	99.9	99.6	99.8	99.9
Index ranges	−24 ≤h ≤25, −25 ≤k ≤25, −31 ≤l ≤31	−17 ≤h ≤23, −23 ≤k ≤23, −30 ≤l ≤30	−37 ≤h ≤36, −32 ≤k ≤31, −34 ≤l ≤34	−35 ≤h ≤35, −31 ≤k ≤31, −33 ≤l ≤33	−35 ≤h ≤35, −32 ≤k ≤32, −32 ≤l ≤32	−35 ≤h ≤34, −31 ≤k ≤32, −32 ≤l ≤27	−35 ≤h ≤35, −30 ≤k ≤32, −33 ≤l ≤23
Reflections collected	88186	43870	226830	217529	196951	136574	144753
Unique reflections	5371	4692	22615	20393	20362	20366	20465
*R*_int_	0.0393	0.0983	0.0822	0.1318	0.1046	0.1401	0.1321
Data/restraints/parameters	5371/78/153	4692/78/141	22615/464/673	20393/372/634	20362/378/649	20366/384/658	20465/378/649
Goodness of fit on F^2^	1.062	1.012	1.154	1.016	1.052	1.066	1.019
*R*_1_ ^a)^ (I >2σ(I)) (Full data)	0.0391	0.1315	0.0802	0.1076	0.0636	0.0942	0.1119
w*R*_2_ ^b)^ (I >2σ(I)) (Full data)	0.1075	0.2825	0.2112	0.1801	0.1157	0.1637	0.2004
Largest difference peak and hole (e/Å^3^)	2.013 and −0.523	2.190 and −3.296	4.928 and −2.408	2.852 and −1.707	1.874 and −0.819	1.790 and −1.306	5.085 and −2.754

^a)^ *R*_1_ = Ʃǁ*F_0_* ǀ−ǀ*F_c_* ǁ/Ʃǀ*F_0_* ǀ.

^b)^ w*R*_2_ = [Ʃ*w*(*F_0_* ^2^−*F_c_* ^2^)^2^/Ʃ*w*(*F_0_* ^2^)^2^]^1/2^.

**Table 2:** Single crystal data and structure refinement parameters for the compounds (CCDC No. 2443922–2443928).

Compound	Tb‐JUB‐1	Dy‐JUB‐1	Ho‐JUB‐1	Er‐JUB‐1	Tm‐JUB‐1	Yb‐JUB‐1	Lu‐JUB‐1
Empirical formula	TbPd_12_As_8_Ba_6_C_56_H_32_O_70_N_2_	DyPd_12_As_8_Ba_6_C_56_H_32_O_68_N_2_	HoPd_12_As_8_Ba_6_C_56_H_32_O_68_N_2_	ErPd_12_As_8_Ba_6_C_56_H_32_O_66_N_2_	TmPd_12_As_8_Ba_6_C_56_H_32_O_70_N_2_	YbPd_12_As_8_Ba_6_C_56_H_32_O_70_N_2_	LuPd_12_As_8_Ba_6_C_56_H_32_O_68_N_2_
Formula weight (g/mol)	4711.95	4683.53	4685.96	4656.29	4721.96	4726.07	4696.00
Crystal system	Orthorhombic	Orthorhombic	Orthorhombic	Orthorhombic	Orthorhombic	Orthorhombic	Orthorhombic
Space group	*Pnma*	*Pnma*	*Pnma*	*Pnma*	*Pnma*	*Pnma*	*Pnma*
*a* (Å)	29.8806(6)	29.6666(17)	29.7663(18)	29.8304(8)	29.8838(8)	29.9556(8)	30.0817(5)
*b* (Å)	27.2882(6)	27.5200(17)	27.3489(17)	27.1366(9)	27.3025(8)	27.1182(8)	26.5474(6)
*c* (Å)	27.8717(6)	27.5151(17)	27.6431(16)	27.7522(8)	27.8663(7)	27.9881(9)	28.0840(6)
α (⁰)	90	90	90	90	90	90	90
ß (⁰)	90	90	90	90	90	90	90
γ (⁰)	90	90	90	90	90	90	90
Volume (Å^3^)	22726.2(8)	22464(2)	22504(2)	22465.3(12)	22736.2(11)	22735.9(12)	22427.6(8)
Z	4	4	4	4	4	4	4
*D*_calc._ (gm/cm^3^)	1.377	1.385	1.383	1.377	1.379	1.381	1.391
Absorption coefficient (mm^−1^)	3.456	3.513	3.526	3.553	3.534	3.555	3.626
F(000)	8636	8576	8580	8520	8652	8656	8596
θ range for data collection (deg)	1.546–25.060	1.560–25.027	1.554–25.027	1.550–25.027	1.546–25.027	1.542 to 24.998	2.413 to 26.000
Completeness to θ*_max_* (%)	99.4	99.8	99.9	99.9	99.7	99.7	99.5
Index ranges	−35 ≤h ≤35, −32 ≤k ≤31, −33 ≤l ≤32	−31 ≤h ≤35, −32 ≤k ≤32, −32 ≤l ≤32	−35 ≤h ≤35, −32 ≤k ≤32, −32 ≤l ≤32	−35 ≤h ≤35, −27 ≤k ≤32, −33 ≤l ≤31	−35 ≤h ≤33, −32 ≤k ≤ 22, −33 ≤l ≤32	−28 ≤h ≤35, −32 ≤k ≤32, −32 ≤l ≤33	−36 ≤h ≤35, −32 ≤k ≤31, −34 ≤l ≤30
Reflections collected	177345	379732	351430	170410	143944	120727	116290
Unique reflections	20488	20277	20311	20288	20495	20428	22256
R_int_	0.1254	0.0508	0.1412	0.0709	0.1126	0.1522	0.0720
Data/restraints/parameters	20488/384/658	20277/378/697	20311/378/649	20288/372/676	20495/384/658	20428/384/658	22256/378/649
Goodness of fit on F^2^	1.014	1.057	1.017	1.009	1.007	1.005	1.157
*R*_1_ ^a)^ (I >2σ(I)) (Full data)	0.0753	0.0507	0.0852	0.0853	0.0796	0.1209	0.1097
w*R*_2_ ^b)^ (I >2σ(I)) (Full data)	0.1341	0.1271	0.1517	0.1782	0.1301	0.1821	0.2391
Largest difference peak and hole (e/Å^3^)	3.237 and −1.459	2.783 and −1.764	3.012 and −2.443	4.437 and −3.110	3.312 and −1.041	5.103 and ‐1.885	4.860 and ‐3.318

^a)^ *R*_1_ = Ʃǁ*F_0_* ǀ‐ǀ*F_c_* ǁ/Ʃǀ*F_0_* ǀ.

^b)^ w*R*_2_ = [Ʃ*w*(*F_0_* ^2^‐*F_c_* ^2^)^2^/Ʃ*w*(*F_0_* ^2^)^2^]^1/2^

**Figure 2:**
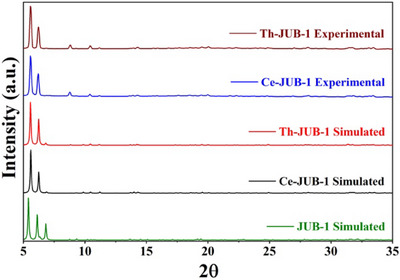
Experimental PXRD patterns of the **Ce‐JUB‐1** and the **Th‐JUB‐1** compared with the simulated PXRD patterns of **Ce‐JUB‐1**, **Th‐JUB‐1**, and **JUB‐1**.

**Figure 3:**
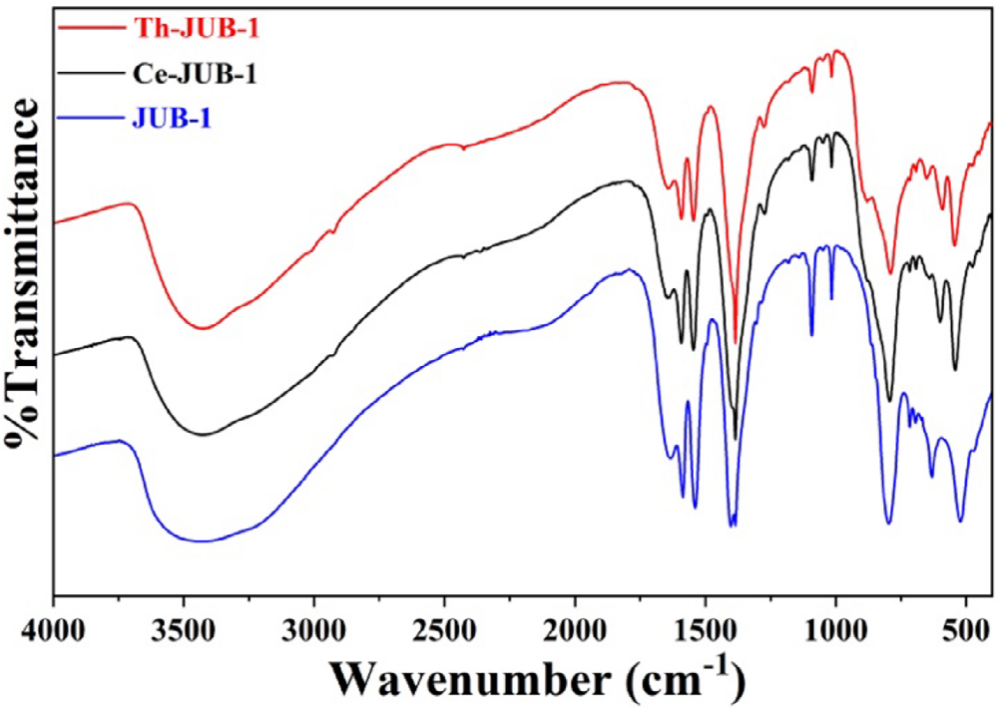
Comparison of the IR spectra of **Ce‐JUB‐1**, **Th‐JUB‐1**, and **JUB‐1**.

Powder X‐ray diffraction (PXRD) experiments performed on the **Ce‐JUB‐1** and the **Th‐JUB‐1** indicated the purity and crystallinity of the samples prepared, as evident from the fact that the PXRD patterns of the as‐prepared samples and the simulated PXRD patterns that were calculated from the single‐crystal X‐ray diffraction (SC‐XRD) data matched well (see Figure 2). It was also observed from PXRD studies that the Pd‐centered **JUB‐1** did not co‐crystallize with the **Ce‐JUB‐1** or the **Th‐JUB‐1**, further reinforcing the purity of the compounds prepared. Fourier transform infrared spectra (FTIR, KBr pellets) were recorded with a Nicolet‐Avatar 370 spectrometer (4000–400 cm^−1^). The IR spectra of **Ce‐JUB‐1**, **Th‐JUB‐1**, all the **Ln‐JUB‐1** as well as of **JUB‐1** ^[^ ^6^ ^]^ appear similar to each other in the region 4000–690 cm^−1^ (albeit with only minor variations). The peaks in this region are as follows: ∼3600–3200 cm^−1^ (s) [ν(O─H) of H_2_O], ∼3100–2800 cm^−1^ (m) [ν(C─H) of phenyl groups and the methyl groups of the lattice cacodylates], ∼2500–2000 cm^−1^ (w) [ν(O─H) of ─AsO_2_H, “A” and “B” bands],^[^ ^8^ ^]^ ∼1640 cm^−1^ (m) [bending vibrations of lattice water molecules], ∼1620–1490 cm^−1^ (s) [overlap of the ν_asymm_ and the ν_symm_ stretching of carboxylate and the “C” band of the [ν(O‐H) of ‐AsO_2_H], ∼1480–1010 cm^−1^ (s) [ν(C─O), ν(C─C), δ(O─H), δ(C─H)], ∼880 and ∼790 cm^−1^ (s) [ν(As─O) of arsonates and arsinates],^[^ ^2^, ^8^ ^]^ and ∼720–690 cm^−1^ (w) [δ(C─C) and δ(C─H)]. The differences in the IR spectra of the **M‐JUB‐1** (M = Ce^III^, Th^IV^, Ln^III^) as compared to that of **JUB‐1** are observed in the region 680–430 cm^−1^, which is the characteristic region for the appearance of the stretching frequencies of the Pd─O bonds as well as of the M─O bonds. As seen in Figures 3 and S3, the three ν(Pd─O) peaks at ∼664 cm^─1^ (w), ∼632 cm^─1^ (m), and ∼521 cm^─1^ (s) observed in the IR spectrum of **JUB‐1**, shift to ∼642 cm^─1^ (w), ∼598 cm^─1^ (m), and ∼542 cm^─1^ (s) for **Ce‐JUB‐1**, to ∼652 cm^─1^ (w), 590 cm^─1^ (m), and ∼544 cm^─1^ (s) for **Th‐JUB‐1**, and to ∼655 cm^─1^ (w), ∼599–619 cm^─1^ (m), and ∼540 cm^─1^ (s) for the other **Ln‐JUB‐1**, indicating that the Ce, Th and the Ln atoms got successfully incorporated into the polyoxopalladate cubic core.^[^ ^2a,c^ ^]^ The peaks for all the compounds in the region 480–440 cm^─1^ correspond to the overlap of the δ(Pd‐O) and the stretching frequencies of the central M─O bonds.^[^ ^9^ ^]^

**Figure 4:**
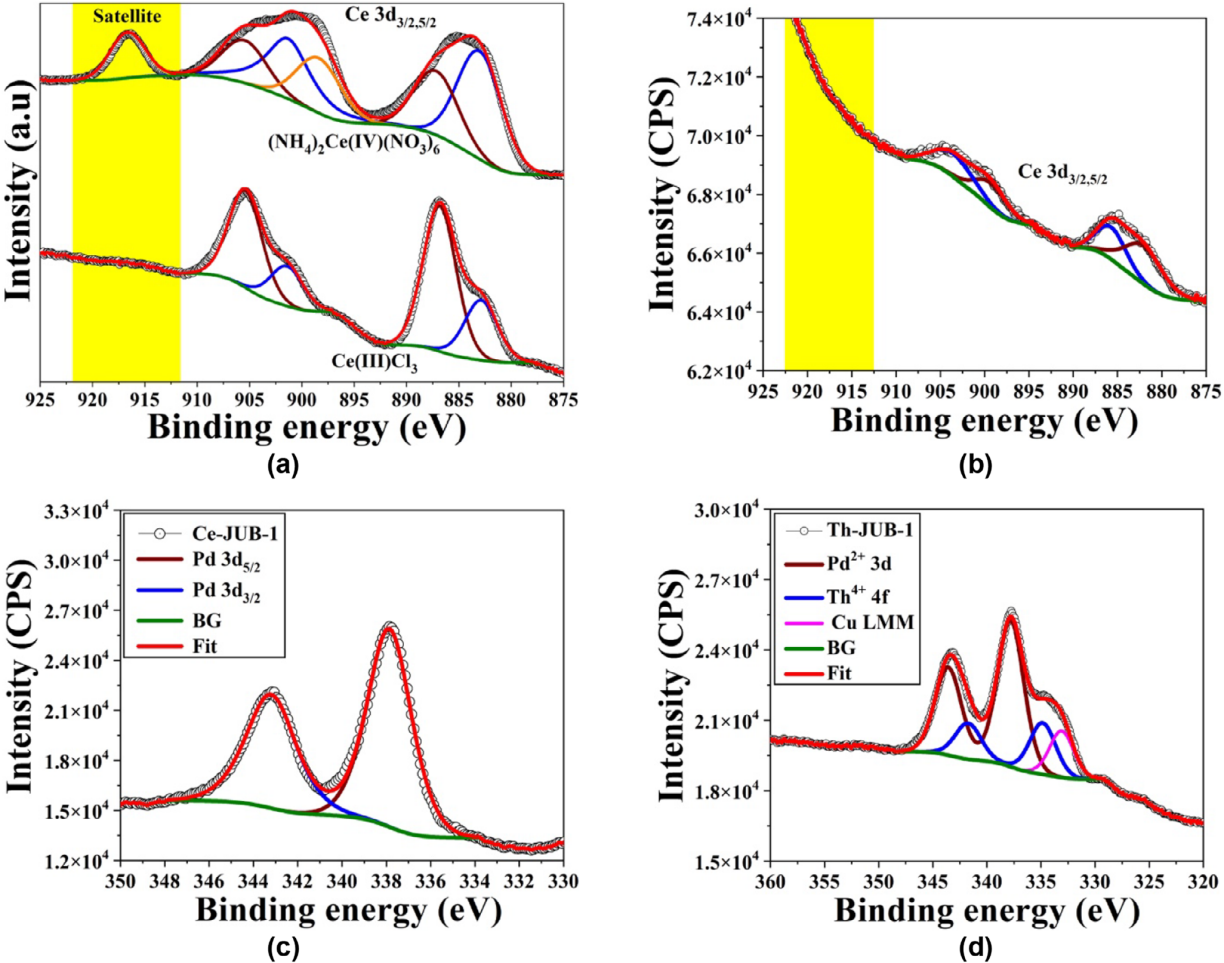
a) X‐ray photoelectron spectra and fits for Ce 3d_5/2_ and 3d_3/2_ multiplets of the cerium‐containing reference materials, CeCl_3_ and (NH_4_)_2_Ce(NO_3_)_6_, and of b) **Ce‐JUB‐1**. The absence of the satellite peak at ∼916.5 eV confirms that the Ce in **Ce‐JUB‐1** is in 3+ oxidation state. c) X‐ray photoelectron spectra and fits for Pd 3d_5/2_ and 3d_3/2_ doublet of **Ce‐JUB‐1**, and d) Pd 3d_5/2_ and 3d_3/2_ doublet as well as Th 4f_7/2_ and 4f_5/2_ doublet of **Th‐JUB‐1**.

X‐ray photoelectron spectroscopy (XPS) measurements were performed on both **Ce‐JUB‐1** and **Th‐JUB‐1** in order to ascertain the oxidation states of Pd, Ce, and Th (Figure 4). Both **Ce‐JUB‐1** and **Th‐JUB‐1** exhibited a Pd 3d_5/2_ band at ∼337 eV, which is typical for Pd in a 2+ oxidation state. The XPS spectra of the reference materials CeCl_3_ and (NH_4_)_2_Ce(NO_3_)_6_ (references for Ce^III^ and Ce^IV^, respectively) are given in Figure 4a, which exhibit the characteristic multiplet peaks of the spin‐orbit split 3d_5/2_ and 3d_3/2_ core in the region 880–910 eV.^[^ ^10^ ^]^ A point to note is that the satellite peak observed at ∼916.5 eV for the (NH_4_)_2_Ce(NO_3_)_6_ is indicative of the presence of Ce^IV^. This peak was found to be absent in the XPS spectrum of **Ce‐JUB‐1** showing that Ce is in 3+ oxidation state (Figure 4b). The XPS spectrum of **Th‐JUB‐1** indicated the characteristic peaks corresponding to the 4f_7/2_ and 4f_5/2_ cores at ∼337.6 and ∼344.0 eV, respectively, confirming the 4+ oxidation state of the Th (Figure 4d).^[^ ^3^, ^11^ ^]^ Peaks corresponding to the Pd 3d_5/2_ and 3d_3/2_ cores were found to be at ∼337 and ∼342 eV, respectively, for both the compounds, which are indicative of Pd in the 2+ oxidation state (Figure 4c,d).^[^ ^3^, ^6^ ^]^

### 3.1 Electrochemical Studies

The electrochemistry of the Ce^III^‐centered POP‐MOF, **Ce‐JUB‐1**, was carried out in aqueous solutions at pH 4. For this purpose, the stability of **Ce‐JUB‐1** was assessed by cyclic voltammetry (CV). The solid **Ce‐JUB‐1** was first immobilized on the surface of the basal plane of the pyrolytic graphite disk (PGB) and the electrochemical response was studied in pH 4.0 (0.5 M H_2_SO_4_–Na_2_SO_4_) buffer solutions.

**Figure 5:**
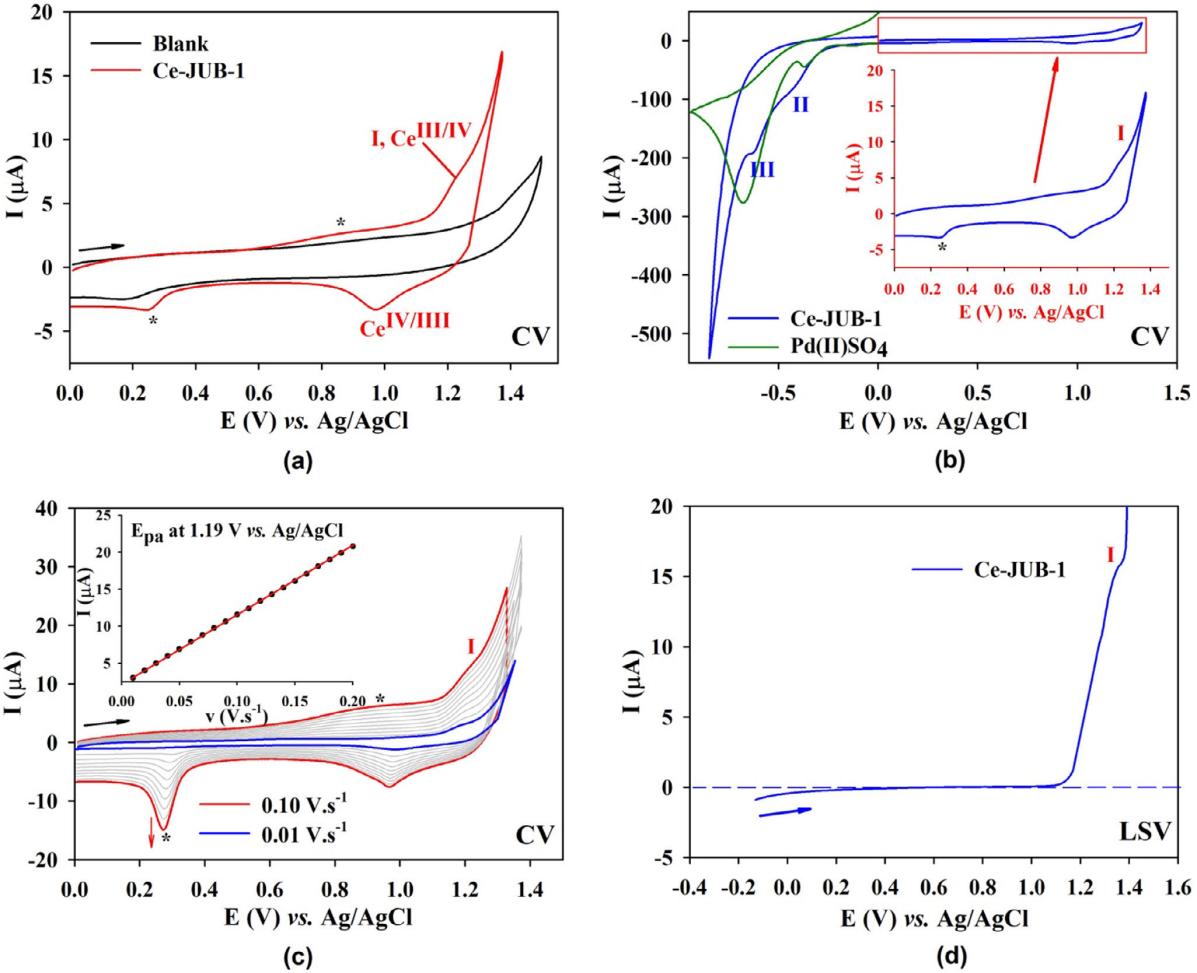
a)–b) Cyclic voltammograms of **Ce‐JUB‐1** immobilized at a PGB electrode (*d* = 2 mm) in a 0.5 M pH 4.0 H_2_SO_4_–Na_2_SO_4_ buffer solution. Scan rate: 20 mV s^─1^. c) Cyclic voltammograms at various scan rate between 0 and 1.325 V. Inset: plots of *i*_pa_ versus *v*. Green curve: CV of 0.3 mM of Pd(II)SO_4_ in a 0.5 M pH 4.0 H_2_SO_4_–Na_2_SO_4_ buffer solution. d) Normal pulse voltammetry (NPV) plot for **Ce‐JUB‐1** measured at pH 4.

Figure 5a features the CVs of **Ce‐JUB‐1** obtained at 20 mV.s^─1^ between 0 and 1.33 V versus Ag/AgCl. The CV of **Ce‐JUB‐1** exhibits a quasi‐reversible oxidation process near 1.19 V versus Ag/AgCl (*E*_pa_ = 1.19 V and E_pc_ = 0.96 V), which may correspond to the Ce(III)/Ce(IV) couple. The peak‐to‐peak separation (Δ*E*_p_) is 230 mV at 0.1 V s^─1^ suggesting not a very rapid electron transfer. It can be explained by the difficult accessibility of the Ce(III) within the **Ce‐JUB‐1** structure, which is not in direct contact with the working electrode, leading to a longer distance of electron transfer. As shown in the inset of Figure 5b, peak current intensities of this wave vary linearly with the scan rate v, as expected for surface‐confined redox processes. Thus, cyclic voltammetry can be repeated without degradation of the **Ce‐JUB‐1** compound upon oxidation. Furthermore, normal pulse voltammetry (NPV) between −0.1 V and +1.4 V versus Ag/AgCl was also measured showing one wave having a positive current suggesting oxidation of Ce(III) to Ce(IV). It must be noted that at the starting of the scan at an applied potential of −0.1 V nearly no current has been measured showing that the initial oxidation state of the Ce is +3.

Nevertheless, the CV of **Ce‐JUB‐1** also exhibits irreversible reduction waves at −0.40 and −0.60 V versus Ag/AgCl as shown in Figure 5c. The cyclic voltammetry of the PdSO_4_ solution in the same conditions exhibits similar waves suggesting irreversible reduction of the Pd(II) atoms forming metallic Pd(0). It must be noted that at the reverse sweep, one additional anodic peak at 0.80 V versus AgCl/Ag appeared. It corresponds to the anodic dissolution peak of Pd(0) and indicates that the irreversible reduction of Pd(II) to Pd(0) leads to decomposition of **Ce‐JUB‐1**.

## 4 Conclusion

In this paper, we report the syntheses, structures, and properties of the first Ce^III^‐centered POP‐MOF, **Ce‐JUB‐1**, as well as the first actinide‐centered POP‐MOF, **Th‐JUB‐1**, along with the isolation of other lanthanide‐centered POP‐MOFs, **Ln‐JUB‐1**. The compounds have been characterized by various solid‐state techniques, such as single‐crystal and powder X‐ray diffraction, IR spectroscopy, and XPS spectroscopy. The oxidation state of the central cerium ion in **Ce‐JUB‐1** as well as the nature of its redox behavior have been ascertained using detailed electrochemical studies. The Ce^III^/Ce^IV^ couple exhibits a quasi‐reversible oxidation process without indicating any degradation of the compound. The discovery of an actinide‐centered POP‐MOF is extremely important as it opens up avenues for the use of POPs as radiopharmaceutical agents for anticancer therapy,^[^ ^7^ ^]^ something that has been shown to be promising in ^224^Ra and ^205/206^Bi‐labeled POPs.^[^ ^4^ ^]^ Work in this area is ongoing in our laboratory.

## Supporting Information

The supporting information includes the detailed instrumental methods employed, additional IR spectral data, as well as additional structure figures. The authors have cited additional references within the Supporting Information.^[12]^

## Conflict of Interests

The authors declare no conflict of interest

## Supporting information



Supporting Information

Supporting Information

## Data Availability

The data that support the findings of this study are available in the supplementary material of this article.
