# Supplementary material for: Lanthanide and Actinide‐Centered Polyoxo‐Noble‐Metalate‐Based Metal−Organic Frameworks
Source: Chem Asian J. 2025 Jul 8;20(18):e00737. doi: 10.1002/asia.202500737 (PMC12450036; doi:10.1002/asia.202500737)

**Electronic Supporting Information**

**Lanthanide and Actinide-Centered Polyoxo-Noble-Metalate-Based Metal−Organic Frameworks**

Saurav Bhattacharya,*^a,b^ Anupam Sarkar,^a^ Tsedenia A. Zewdie,^a^ Alina J. George,^a^ Samer Dawoud,^c^ Talha Nisar,^a^ Christian J. Schürmann,^d^ Veit Wagner,^a^ Laurent Ruhlmann,^c^ and Ulrich Kortz*^a^

^a^School of Science, Constructor University

Campus Ring 1, 28759 Bremen, Germany

^b^Department of Chemistry, Birla Institute of Technology and Science, Pilani,

K K Birla Goa Campus,

Zuarinagar, Sancoale, Goa-403726, India

^c^ Institute of Chemistry (UMR au CNRS n°7177), University of Strasbourg, 4, rue Blaise Pascal, Strasbourg, France

^d^Rigaku Europe SE

Hugenottenallee 167, 63263 Neu-Isenburg, Germany

**1. Additional Experimental Information**

**1.1 Instrumentation**

The elemental analyses for the elements Ce, Th, Pd, As, Ba, Na, and other lanthanides were performed at Zentrallabor Chemische Analytik in Technische Universität Hamburg, Eißendorfer Str. 38, 21073 Hamburg. The CHN analyses were performed at Analytische Laboratorien, Industriepark Kaiserau (Haus Heidbruch), 51789 Lindlar, Germany. Fourier Transform Infrared spectra (FTIR, KBr pellets) were recorded on a Nicolet-Avatar 370 spectrometer (4000 – 400 cm^-1^). Powder X-ray diffraction (PXRD) patterns were collected on a Rigaku Synergy-S system by mounting a few micrograms of the samples on a 200 μm nylon loop. The measurement was performed using a Cu (1.54 Å) PhotonJet source with automatic divergence control and reduced divergence of 1.8 mrad and a HyPix6000HE detector at a distance of 120mm. During the measurement, the sample orientation was randomized by Gandolfi scans. The 1D diffraction pattern was extracted by integrating frames from three different 2θ positions in CrysAlisPro (version 41.116a, Rigaku Oxford Diffraction, Wroclaw, Poland). The oxidation states of Ce, Th and Pd in the two compounds were ascertained using X-ray photoelectron spectroscopy (XPS) measurements. The samples were dispersed in acetone and spin-coated onto the as prepared substrates at a rotation speed of 1000 rpm. The ultra-high vacuum vessel, which had a vacuum pressure of ~1 x 10^–8^ mbar, was equipped with a water-cooled X-ray gun with a double Mg/Al anode (Specs XR 50) and a hemispherical electron analyzer (Specs Phoibos 100). Mg Kα1,2 radiation (E = 1253.6 eV) was used as a source of excitation. The photoelectrons were detected in the large area lens mode and fixed analyzer transmission at a pass energy of 50 eV. The measured data were analyzed using the CASA-XPS software. The positions of the peaks were in the expected regions (see main text for details). For single-crystal X-ray diffraction experiments, the crystals were mounted on a Hampton cryoloop or MiTeGen mount in light oil for data collection at 100 K on Bruker D8 SMART APEX II CCD (**Th-JUB-1** and other **Ln-JUB-1**) or Rigaku XtaLAB Synergy – S (**Ce-JUB-1**) kappa geometry diffractometers. Data were measured using ω-scans of 0.5° per frame using Mo Kα radiation (λ = 0.71073 Å) and processed with APEX III software package^12a^ and CrysAlisPro software packages, respectively. Routine Lorentz and polarization corrections were applied, and absorption corrections were performed using SADABS^12b^ or SCALE3 ABSPACK. Space group assignments were based upon systematic absences, E-statistics, and successful refinement of the structures. The structures were solved by direct methods with the aid of successive difference Fourier maps and were refined against all data using SHELXL-2019.^12c^ Refinements were conducted by full-matrix least squares against |F| using all data. The H-atoms of the phenyl groups were placed in calculated positions and then refined. In the final refinement, all non-hydrogen atoms were refined anisotropically. The framework guest molecules residing in the channels were either too disordered or could not be located by XRD and, therefore, the corresponding disagreeable reflections were removed by the OMIT and SQUEEZE command in PLATON.^12d^ The images of the crystal structures were generated by Diamond, version 5.0.2 (software copyright 1997-2024, Crystal Impact GbR, Bonn, Germany). The crystallographic data and structure refinement parameters are summarized in Table S1. The cif files are provided free of charge by the Cambridge Crystallographic Data Centre (CCDC 2443915-2443928).

Voltammetric data have been recorded with a standard three-electrode system using a PARSTAT 2273 potentiostat using a conventional three-electrode setup. Potentials are quoted against an Ag/AgCl electrode. The counter electrode was a platinum gauze of large surface area. All experiments were performed at room temperature. The working electrode was basal plane pyrolytic graphite disk (PGB, basal plane, Pine, Ø = 5 mm) or a glassy carbon electrode (Ø=3mm), the counter electrode was platinum wire, and the reference electrode was an Ag/AgCl electrode connected through a salt bridge. The working cell was surrounded by a grounded Faraday cage and all studies were carried out at room temperature and under an argon flow. Ultrapure water (Millipore, 18.2 MΩ.cm^-1^, 25 °C) was used to prepare all electrolyte solutions. The composition and pH of the media used for both the electrochemical experiments and the stability studies by spectrophotometry were as follows: 0.5 M Na_2_SO_4_ + H_2_SO_4_ (pH 4).

The solutions were deaerated thoroughly by bubbling argon through the solution and kept under argon atmosphere during the whole experiment. The basal plane of the pyrolytic graphite electrode (PGB) was cleaned before each measurement according to the following procedure: polishing on a micro-cloth polishing pad with diamond paste (DP-paste M, 1, 3 and 6 μm particle sizes); washing with ultrapure water; sonication in an ultrasonic bath for 5 min.

Electrode modification consisted of depositing an appropriate amount of powder of each material on the PGB surface (Ø=5mm). The glassy carbon (Le Carbone-Lorraine) electrode (Ø = 5 mm) was also used to study PdSO_4_ in aqueous solution and was cleaned before each measurement according to the same procedure as described for the basal plane of the pyrolytic graphite electrode.

[12] (a) APEX suite of crystallographic software, APEX 3, version 2015.5-2, Bruker AXS Inc., Madison, Wisconsin, USA, **2015**; (b) SAINT, Version 7.56a and SADABS Version 2008/1, Bruker AXS Inc., Madison, Wisconsin, USA, **2008**; (c) G. M. Sheldrick, Acta Cryst. **2015**, C71, 3-8; (d) A. L. Spek, PLATON, A Multipurpose Crystallographic Tool, Utrecht University, Utrecht, The Netherlands, **2010**.

**Table S1.** Selected bond distances obtained from single-crystal X-ray diffraction studies

| **Ce-JUB-1** | |
| --- | --- |
| **Bond** | **Length (Å)** |
| Ce(1)-O(1) | 2.348(3) |
| Ce(1)-O(1)#1 | 2.348(3) |
| Ce(1)-O(1)#2 | 2.348(3) |
| Ce(1)-O(1)#3 | 2.348(3) |
| Ce(1)-O(1)#4 | 2.348(3) |
| Ce(1)-O(1)#5 | 2.348(3) |
| Ce(1)-O(1)#6 | 2.348(3) |
| Ce(1)-O(1)#7 | 2.348(3) |
| Pd(1)-O(1) | 1.993(3) |
| Pd(1)-O(1)#4 | 1.993(3) |
| Pd(1)-O(2) | 1.996(3) |
| Pd(1)-O(2)#4 | 1.996(3) |
| Pd(2)-O(1) | 1.993(3) |
| Pd(2)-O(1)#7 | 1.996(3) |
| Pd(2)-O(3)#7 | 2.026(3) |
| Pd(2)-O(4) | 2.034(3) |
| Symmetry transformations used to generate equivalent atoms:  #1: -x+2,-y+2,-z+1; #2: -y+2,x,-z+1; #3: y,-x+2,z; #4: x,y,-z+1; #5: -x+2,-y+2,z; #6: y,-x+2,-z+1; #7: -y+2,x,z. | |

| **Th-JUB-1** | |
| --- | --- |
| **Bond** | **Length (Å)** |
| Th(1)-O(1) | 2.402(10) |
| Th(1)-O(1)#1 | 2.402(10) |
| Th(1)-O(1)#2 | 2.402(10) |
| Th(1)-O(1)#3 | 2.402(10) |
| Th(1)-O(1)#4 | 2.402(10) |
| Th(1)-O(1)#5 | 2.402(10) |
| Th(1)-O(1)#6 | 2.402(10) |
| Th(1)-O(1)#7 | 2.402(10) |
| Pd(1)-O(2) | 1.979(12) |
| Pd(1)-O(2)#4 | 1.979(12) |
| Pd(1)-O(1)#6 | 1.997(10) |
| Pd(1)-O(1)#3 | 1.997(10) |
| Pd(2)-O(1) | 2.003(10) |
| Pd(2)-O(1)#3 | 2.015(10) |
| Pd(2)-O(3) | 2.023(12) |
| Pd(2)-O(4) | 2.037(12) |
| Symmetry transformations used to generate equivalent atoms:  #1: -x,-y,-z+1; #2: -y,x,-z+1; #3: y,-x,z; #4: x,y,-z+1; #5: -x,-y,z; #6: y,-x,-z+1; #7: -y,x,z. | |

| **Pr-JUB-1** | | | |
| --- | --- | --- | --- |
| **Bond** | **Length (Å)** | **Bond** | **Length (Å)** |
| Pr(1)-O(2) | 2.413(5) | Pd(4)-O(10)#1 | 1.976(5) |
| Pr(1)-O(2)#1 | 2.413(5) | Pd(4)-O(10) | 1.976(5) |
| Pr(1)-O(1) | 2.421(5) | Pd(4)-O(2)#1 | 2.017(5) |
| Pr(1)-O(1)#1 | 2.421(5) | Pd(4)-O(2) | 2.017(5) |
| Pr(1)-O(3) | 2.423(5) | Pd(5)-O(2) | 1.993(5) |
| Pr(1)-O(3)#1 | 2.423(5) | Pd(5)-O(3) | 2.001(5) |
| Pr(1)-O(4) | 2.431(5) | Pd(5)-O(11) | 2.017(5) |
| Pr(1)-O(4)#1 | 2.431(5) | Pd(5)-O(12) | 2.024(5) |
| Pd(1)-O(1)#1 | 1.988(5) | Pd(6)-O(4) | 1.979(5) |
| Pd(1)-O(1) | 1.988(5) | Pd(6)-O(2) | 1.989(5) |
| Pd(1)-O(5) | 1.993(6) | Pd(6)-O(13) | 2.017(5) |
| Pd(1)-O(5)#1 | 1.993(6) | Pd(6)-O(14) | 2.037(5) |
| Pd(2)-O(3) | 1.996(5) | Pd(7)-O(3) | 2.003(5) |
| Pd(2)-O(1) | 2.010(5) | Pd(7)-O(3)#1 | 2.003(5) |
| Pd(2)-O(7) | 2.031(5) | Pd(7)-O(15) | 2.012(5) |
| Pd(2)-O(6) | 2.043(5) | Pd(7)-O(15)#1 | 2.012(5) |
| Pd(3)-O(4) | 1.989(5) | Pd(8)-O(4)#1 | 1.997(5) |
| Pd(3)-O(1) | 1.990(5) | Pd(8)-O(4) | 1.998(5) |
| Pd(3)-O(8) | 2.019(5) | Pd(8)-O(16)#1 | 2.010(6) |
| Pd(3)-O(9) | 2.035(5) | Pd(8)-O(16) | 2.010(6) |
| Symmetry transformations used to generate equivalent atoms: #1: x,-y+3/2,z. | | | |

| **Nd-JUB-1** | | | |
| --- | --- | --- | --- |
| **Bond** | **Length (Å)** | **Bond** | **Length (Å)** |
| Nd(1)-O(2)#1 | 2.388(8) | Pd(4)-O(10)#1 | 2.011(9) |
| Nd(1)-O(2) | 2.388(8) | Pd(4)-O(10) | 2.011(9) |
| Nd(1)-O(3) | 2.391(8) | Pd(4)-O(2)#1 | 2.013(8) |
| Nd(1)-O(3)#1 | 2.391(8) | Pd(4)-O(2) | 2.013(8) |
| Nd(1)-O(1) | 2.396(8) | Pd(5)-O(2)#1 | 2.006(7) |
| Nd(1)-O(1)#1 | 2.396(8) | Pd(5)-O(3) | 2.017(8) |
| Nd(1)-O(4)#1 | 2.396(8) | Pd(5)-O(11) | 2.032(8) |
| Nd(1)-O(4) | 2.396(8) | Pd(5)-O(12)#1 | 2.039(8) |
| Pd(1)-O(5) | 1.997(8) | Pd(6)-O(3) | 1.995(7) |
| Pd(1)-O(5)#1 | 1.997(8) | Pd(6)-O(3)#1 | 1.995(7) |
| Pd(1)-O(1)#1 | 2.000(8) | Pd(6)-O(13) | 2.002(9) |
| Pd(1)-O(1) | 2.000(8) | Pd(6)-O(13)#1 | 2.002(9) |
| Pd(2)-O(2)#1 | 2.005(7) | Pd(7)-O(3)#1 | 1.990(8) |
| Pd(2)-O(1) | 2.009(7) | Pd(7)-O(4) | 1.991(7) |
| Pd(2)-O(7) | 2.028(8) | Pd(7)-O(14) | 2.005(9) |
| Pd(2)-O(6) | 2.032(8) | Pd(7)-O(15)#1 | 2.026(9) |
| Pd(3)-O(4)#1 | 1.993(7) | Pd(8)-O(4) | 2.000(7) |
| Pd(3)-O(1) | 1.995(8) | Pd(8)-O(4)#1 | 2.000(7) |
| Pd(3)-O(8) | 2.022(8) | Pd(8)-O(16) | 2.017(8) |
| Pd(3)-O(9) | 2.024(8) | Pd(8)-O(16)#1 | 2.017(8) |
| Symmetry transformations used to generate equivalent atoms: #1: x,-y+1/2,z. | | | |

| **Sm-JUB-1** | | | |
| --- | --- | --- | --- |
| **Bond** | **Length (Å)** | **Bond** | **Length (Å)** |
| Sm(1)-O(2)#1 | 2.378(4) | Pd(4)-O(2)#1 | 2.000(4) |
| Sm(1)-O(2) | 2.378(4) | Pd(4)-O(2) | 2.000(4) |
| Sm(1)-O(1) | 2.379(4) | Pd(4)-O(10)#1 | 2.020(5) |
| Sm(1)-O(1)#1 | 2.379(4) | Pd(4)-O(10) | 2.020(5) |
| Sm(1)-O(3)#1 | 2.382(4) | Pd(5)-O(2) | 1.999(4) |
| Sm(1)-O(3) | 2.382(4) | Pd(5)-O(3) | 2.006(4) |
| Sm(1)-O(4) | 2.403(4) | Pd(5)-O(11)#1 | 2.030(4) |
| Sm(1)-O(4)#1 | 2.403(4) | Pd(5)-O(12) | 2.043(4) |
| Pd(1)-O(1) | 2.006(4) | Pd(6)-O(4) | 1.987(4) |
| Pd(1)-O(1)#1 | 2.006(4) | Pd(6)-O(2) | 1.995(4) |
| Pd(1)-O(5)#1 | 2.024(4) | Pd(6)-O(13) | 1.999(5) |
| Pd(1)-O(5) | 2.024(4) | Pd(6)-O(14) | 2.027(4) |
| Pd(2)-O(1) | 1.987(4) | Pd(7)-O(3) | 1.998(4) |
| Pd(2)-O(3)#1 | 2.013(4) | Pd(7)-O(3)#1 | 1.998(4) |
| Pd(2)-O(7) | 2.029(4) | Pd(7)-O(15)#1 | 2.033(4) |
| Pd(2)-O(6)#1 | 2.048(4) | Pd(7)-O(15) | 2.033(4) |
| Pd(3)-O(4)#1 | 1.986(4) | Pd(8)-O(4)#1 | 1.998(4) |
| Pd(3)-O(1) | 2.011(4) | Pd(8)-O(4) | 1.998(4) |
| Pd(3)-O(8)#1 | 2.020(4) | Pd(8)-O(16) | 2.023(5) |
| Pd(3)-O(9)#1 | 2.036(4) | Pd(8)-O(16)#1 | 2.023(5) |
| Symmetry transformations used to generate equivalent atoms: #1: x,-y+3/2,z | | | |

| **Eu-JUB-1** | | | |
| --- | --- | --- | --- |
| **Bond** | **Length (Å)** | **Bond** | **Length (Å)** |
| Eu(1)-O(2)#1 | 2.372(6) | Pd(4)-O(1)#1 | 2.009(6) |
| Eu(1)-O(2) | 2.372(6) | Pd(4)-O(1) | 2.009(6) |
| Eu(1)-O(3)#1 | 2.374(6) | Pd(4)-O(10)#1 | 2.013(7) |
| Eu(1)-O(3) | 2.374(6) | Pd(4)-O(10) | 2.014(7) |
| Eu(1)-O(1) | 2.374(6) | Pd(5)-O(3) | 1.989(7) |
| Eu(1)-O(1)#1 | 2.375(6) | Pd(5)-O(2) | 1.998(6) |
| Eu(1)-O(4)#1 | 2.400(6) | Pd(5)-O(11)#1 | 2.030(7) |
| Eu(1)-O(4) | 2.400(6) | Pd(5)-O(12) | 2.042(6) |
| Pd(1)-O(2) | 1.990(6) | Pd(6)-O(3) | 2.000(6) |
| Pd(1)-O(2)#1 | 1.990(6) | Pd(6)-O(3)#1 | 2.000(6) |
| Pd(1)-O(5) | 2.033(7) | Pd(6)-O(13) | 2.036(7) |
| Pd(1)-O(5)#1 | 2.033(7) | Pd(6)-O(13)#1 | 2.036(7) |
| Pd(2)-O(1) | 1.976(7) | Pd(7)-O(4) | 1.987(6) |
| Pd(2)-O(2)#1 | 2.016(6) | Pd(7)-O(3) | 1.988(7) |
| Pd(2)-O(7) | 2.023(7) | Pd(7)-O(14) | 2.005(7) |
| Pd(2)-O(6) | 2.045(6) | Pd(7)-O(15) | 2.014(7) |
| Pd(3)-O(4)#1 | 1.986(6) | Pd(8)-O(4)#1 | 1.981(7) |
| Pd(3)-O(1) | 2.007(7) | Pd(8)-O(4) | 1.981(7) |
| Pd(3)-O(8) | 2.013(7) | Pd(8)-O(16) | 2.016(7) |
| Pd(3)-O(9)#1 | 2.027(7) | Pd(8)-O(16)#1 | 2.016(7) |
| Symmetry transformations used to generate equivalent atoms: #1: x,-y+1/2,z. | | | |

| **Gd-JUB-1** | | | |
| --- | --- | --- | --- |
| **Bond** | **Length (Å)** | **Bond** | **Length (Å)** |
| Gd(1)-O(1)#1 | 2.352(8) | Pd(4)-O(2) | 1.972(7) |
| Gd(1)-O(1) | 2.352(8) | Pd(4)-O(2)#1 | 1.972(7) |
| Gd(1)-O(4) | 2.354(7) | Pd(4)-O(10) | 2.008(8) |
| Gd(1)-O(4)#1 | 2.354(7) | Pd(4)-O(10)#1 | 2.008(8) |
| Gd(1)-O(2) | 2.360(7) | Pd(5)-O(3)#1 | 1.976(7) |
| Gd(1)-O(2)#1 | 2.360(7) | Pd(5)-O(2) | 1.991(8) |
| Gd(1)-O(3) | 2.378(8) | Pd(5)-O(12)#1 | 2.020(8) |
| Gd(1)-O(3)#1 | 2.378(8) | Pd(5)-O(11)#1 | 2.042(8) |
| Pd(1)-O(1) | 1.996(8) | Pd(6)-O(3)#1 | 1.977(8) |
| Pd(1)-O(1)#1 | 1.996(8) | Pd(6)-O(3) | 1.977(8) |
| Pd(1)-O(5) | 2.020(9) | Pd(6)-O(13)#1 | 2.003(8) |
| Pd(1)-O(5)#1 | 2.020(9) | Pd(6)-O(13) | 2.003(8) |
| Pd(2)-O(1)#1 | 2.008(7) | Pd(7)-O(4) | 1.979(8) |
| Pd(2)-O(2) | 2.011(8) | Pd(7)-O(3) | 1.993(7) |
| Pd(2)-O(7) | 2.033(9) | Pd(7)-O(15)#1 | 2.030(8) |
| Pd(2)-O(6)#1 | 2.049(7) | Pd(7)-O(14) | 2.031(8) |
| Pd(3)-O(1) | 1.989(7) | Pd(8)-O(4) | 2.003(8) |
| Pd(3)-O(4) | 1.991(8) | Pd(8)-O(4)#1 | 2.004(8) |
| Pd(3)-O(9)#1 | 2.033(8) | Pd(8)-O(16)#1 | 2.025(8) |
| Pd(3)-O(8)#1 | 2.037(8) | Pd(8)-O(16) | 2.025(8) |
| Symmetry transformations used to generate equivalent atoms: #1: x,-y+1/2,z. | | | |

| **Tb-JUB-1** | | | |
| --- | --- | --- | --- |
| **Bond** | **Length (Å)** | **Bond** | **Length (Å)** |
| Tb(1)-O(1)#1 | 2.350(5) | Pd(4)-O(2)#1 | 2.003(5) |
| Tb(1)-O(1) | 2.350(5) | Pd(4)-O(2) | 2.003(5) |
| Tb(1)-O(2)#1 | 2.355(5) | Pd(4)-O(10) | 2.043(5) |
| Tb(1)-O(2) | 2.355(5) | Pd(4)-O(10)#1 | 2.043(5) |
| Tb(1)-O(4) | 2.356(5) | Pd(5)-O(2) | 1.987(5) |
| Tb(1)-O(4)#1 | 2.356(5) | Pd(5)-O(4) | 1.991(5) |
| Tb(1)-O(3) | 2.371(5) | Pd(5)-O(12)#1 | 2.039(6) |
| Tb(1)-O(3)#1 | 2.371(5) | Pd(5)-O(11) | 2.051(5) |
| Pd(1)-O(1) | 1.995(5) | Pd(6)-O(3) | 1.994(5) |
| Pd(1)-O(1)#1 | 1.995(5) | Pd(6)-O(3)#1 | 1.994(5) |
| Pd(1)-O(5)#1 | 2.015(5) | Pd(6)-O(13)#1 | 2.017(6) |
| Pd(1)-O(5) | 2.015(5) | Pd(6)-O(13) | 2.017(6) |
| Pd(2)-O(1) | 2.000(5) | Pd(7)-O(3) | 1.977(5) |
| Pd(2)-O(2)#1 | 2.009(5) | Pd(7)-O(4) | 1.987(5) |
| Pd(2)-O(6) | 2.025(6) | Pd(7)-O(14) | 2.022(5) |
| Pd(2)-O(7)#1 | 2.056(5) | Pd(7)-O(15) | 2.022(5) |
| Pd(3)-O(3)#1 | 1.985(5) | Pd(8)-O(4)#1 | 2.001(5) |
| Pd(3)-O(1) | 1.996(5) | Pd(8)-O(4) | 2.001(5) |
| Pd(3)-O(8)#1 | 2.027(5) | Pd(8)-O(16) | 2.017(5) |
| Pd(3)-O(9)#1 | 2.033(5) | Pd(8)-O(16)#1 | 2.017(5) |
| Symmetry transformations used to generate equivalent atoms: #1: x,-y+3/2,z. | | | |

| **Dy-JUB-1** | | | |
| --- | --- | --- | --- |
| **Bond** | **Length (Å)** | **Bond** | **Length (Å)** |
| Dy(1)-O(2) | 2.340(4) | Pd(4)-O(2) | 1.991(4) |
| Dy(1)-O(2)#1 | 2.340(4) | Pd(4)-O(2)#1 | 1.991(4) |
| Dy(1)-O(4) | 2.343(4) | Pd(4)-O(10) | 2.011(4) |
| Dy(1)-O(4)#1 | 2.343(4) | Pd(4)-O(10)#1 | 2.011(4) |
| Dy(1)-O(1) | 2.343(4) | Pd(5)-O(3) | 1.991(4) |
| Dy(1)-O(1)#1 | 2.343(4) | Pd(5)-O(2) | 2.000(4) |
| Dy(1)-O(3)#1 | 2.351(4) | Pd(5)-O(11)#1 | 2.020(4) |
| Dy(1)-O(3) | 2.351(4) | Pd(5)-O(12) | 2.038(4) |
| Pd(1)-O(1)#1 | 1.998(4) | Pd(6)-O(3) | 1.977(4) |
| Pd(1)-O(1) | 1.998(4) | Pd(6)-O(3)#1 | 1.977(4) |
| Pd(1)-O(5) | 2.031(4) | Pd(6)-O(13) | 2.013(4) |
| Pd(1)-O(5)#1 | 2.031(4) | Pd(6)-O(13)#1 | 2.013(4) |
| Pd(2)-O(1)#1 | 1.994(4) | Pd(7)-O(4) | 1.987(4) |
| Pd(2)-O(4) | 1.996(4) | Pd(7)-O(3)#1 | 1.988(4) |
| Pd(2)-O(6) | 2.027(4) | Pd(7)-O(14) | 2.014(4) |
| Pd(2)-O(7) | 2.048(4) | Pd(7)-O(15)#1 | 2.028(4) |
| Pd(3)-O(2) | 1.991(4) | Pd(8)-O(4) | 1.985(4) |
| Pd(3)-O(1) | 2.002(4) | Pd(8)-O(4)#1 | 1.985(4) |
| Pd(3)-O(9)#1 | 2.034(4) | Pd(8)-O(16)#1 | 2.021(4) |
| Pd(3)-O(8)#1 | 2.043(4) | Pd(8)-O(16) | 2.021(4) |
| Symmetry transformations used to generate equivalent atoms: #1: x,-y+1/2,z. | | | |

| **Ho-JUB-1** | | | |
| --- | --- | --- | --- |
| **Bond** | **Length (Å)** | **Bond** | **Length (Å)** |
| Ho(1)-O(1)#1 | 2.330(6) | Pd(4)-O(2)#1 | 1.990(6) |
| Ho(1)-O(1) | 2.330(6) | Pd(4)-O(2) | 1.991(6) |
| Ho(1)-O(3) | 2.336(6) | Pd(4)-O(10)#1 | 2.024(6) |
| Ho(1)-O(3)#1 | 2.336(6) | Pd(4)-O(10) | 2.024(6) |
| Ho(1)-O(2) | 2.339(6) | Pd(5)-O(3) | 1.991(6) |
| Ho(1)-O(2)#1 | 2.339(6) | Pd(5)-O(2) | 2.002(6) |
| Ho(1)-O(4)#1 | 2.354(6) | Pd(5)-O(11) | 2.024(6) |
| Ho(1)-O(4) | 2.354(6) | Pd(5)-O(12) | 2.041(6) |
| Pd(1)-O(1) | 1.987(6) | Pd(6)-O(3) | 1.990(6) |
| Pd(1)-O(1)#1 | 1.987(6) | Pd(6)-O(3)#1 | 1.990(6) |
| Pd(1)-O(5)#1 | 2.017(7) | Pd(6)-O(13) | 2.017(6) |
| Pd(1)-O(5) | 2.017(7) | Pd(6)-O(13)#1 | 2.017(6) |
| Pd(2)-O(2)#1 | 1.993(6) | Pd(7)-O(4) | 1.981(6) |
| Pd(2)-O(1) | 1.996(6) | Pd(7)-O(3) | 1.984(6) |
| Pd(2)-O(7)#1 | 2.025(6) | Pd(7)-O(14) | 2.017(6) |
| Pd(2)-O(6) | 2.037(6) | Pd(7)-O(15) | 2.043(6) |
| Pd(3)-O(4)#1 | 1.986(6) | Pd(8)-O(4)#1 | 1.977(6) |
| Pd(3)-O(1) | 1.996(6) | Pd(8)-O(4) | 1.977(6) |
| Pd(3)-O(8) | 2.027(6) | Pd(8)-O(16) | 2.000(6) |
| Pd(3)-O(9)#1 | 2.032(6) | Pd(8)-O(16)#1 | 2.001(6) |
| Symmetry transformations used to generate equivalent atoms: #1: x,-y+1/2,z. | | | |

| **Er-JUB-1** | | | |
| --- | --- | --- | --- |
| **Bond** | **Length (Å)** | **Bond** | **Length (Å)** |
| Er(1)-O(3) | 2.317(6) | Pd(4)-O(2) | 1.985(6) |
| Er(1)-O(3)#1 | 2.317(6) | Pd(4)-O(2)#1 | 1.985(6) |
| Er(1)-O(4) | 2.327(6) | Pd(4)-O(10)#1 | 1.999(7) |
| Er(1)-O(4)#1 | 2.327(6) | Pd(4)-O(10) | 1.999(7) |
| Er(1)-O(1) | 2.327(6) | Pd(5)-O(2) | 1.978(6) |
| Er(1)-O(1)#1 | 2.327(6) | Pd(5)-O(4) | 1.988(6) |
| Er(1)-O(2)#1 | 2.330(6) | Pd(5)-O(11) | 2.020(7) |
| Er(1)-O(2) | 2.330(6) | Pd(5)-O(12)#1 | 2.030(7) |
| Pd(1)-O(1) | 1.989(6) | Pd(6)-O(4) | 1.984(6) |
| Pd(1)-O(3)#1 | 2.002(6) | Pd(6)-O(4)#1 | 1.984(6) |
| Pd(1)-O(6) | 2.031(7) | Pd(6)-O(13) | 2.018(7) |
| Pd(1)-O(5) | 2.037(6) | Pd(6)-O(13)#1 | 2.018(7) |
| Pd(2)-O(1)#1 | 1.984(6) | Pd(7)-O(4) | 1.988(6) |
| Pd(2)-O(1) | 1.984(6) | Pd(7)-O(3) | 1.998(6) |
| Pd(2)-O(7)#1 | 2.000(7) | Pd(7)-O(14)#1 | 2.043(7) |
| Pd(2)-O(7) | 2.000(7) | Pd(7)-O(15) | 2.046(6) |
| Pd(3)-O(2)#1 | 1.987(6) | Pd(8)-O(3) | 1.991(6) |
| Pd(3)-O(1) | 2.000(6) | Pd(8)-O(3)#1 | 1.991(6) |
| Pd(3)-O(8) | 2.013(6) | Pd(8)-O(16)#1 | 2.031(7) |
| Pd(3)-O(9) | 2.022(7) | Pd(8)-O(16) | 2.031(7) |
| Symmetry transformations used to generate equivalent atoms: #1: x,-y+1/2,z. | | | |

| **Tm-JUB-1** | | | |
| --- | --- | --- | --- |
| **Bond** | **Length (Å)** | **Bond** | **Length (Å)** |
| Tm(1)-O(2) | 2.319(5) | Pd(4)-O(2) | 1.993(5) |
| Tm(1)-O(2)#1 | 2.319(5) | Pd(4)-O(2)#1 | 1.993(5) |
| Tm(1)-O(1)#1 | 2.322(5) | Pd(4)-O(10)#1 | 2.049(5) |
| Tm(1)-O(1) | 2.322(5) | Pd(4)-O(10) | 2.049(5) |
| Tm(1)-O(3) | 2.333(5) | Pd(5)-O(3) | 1.987(5) |
| Tm(1)-O(3)#1 | 2.333(5) | Pd(5)-O(2) | 2.001(5) |
| Tm(1)-O(4) | 2.348(5) | Pd(5)-O(11)#1 | 2.042(5) |
| Tm(1)-O(4)#1 | 2.348(5) | Pd(5)-O(12) | 2.048(5) |
| Pd(1)-O(1)#1 | 2.000(5) | Pd(6)-O(3) | 2.002(5) |
| Pd(1)-O(1) | 2.000(5) | Pd(6)-O(3)#1 | 2.002(5) |
| Pd(1)-O(5) | 2.008(5) | Pd(6)-O(13) | 2.013(6) |
| Pd(1)-O(5)#1 | 2.008(5) | Pd(6)-O(13)#1 | 2.013(6) |
| Pd(2)-O(1) | 1.995(5) | Pd(7)-O(3) | 1.982(5) |
| Pd(2)-O(2)#1 | 2.002(5) | Pd(7)-O(4) | 1.983(5) |
| Pd(2)-O(7)#1 | 2.044(5) | Pd(7)-O(14)#1 | 2.013(5) |
| Pd(2)-O(6)#1 | 2.051(5) | Pd(7)-O(15) | 2.040(5) |
| Pd(3)-O(4)#1 | 1.968(5) | Pd(8)-O(4)#1 | 1.995(5) |
| Pd(3)-O(1) | 1.989(5) | Pd(8)-O(4) | 1.995(5) |
| Pd(3)-O(8)#1 | 2.022(5) | Pd(8)-O(16) | 2.020(6) |
| Pd(3)-O(9)#1 | 2.035(5) | Pd(8)-O(16)#1 | 2.020(6) |
| Symmetry transformations used to generate equivalent atoms: #1: x,-y+1/2,z. | | | |

| **Yb-JUB-1** | | | |
| --- | --- | --- | --- |
| **Bond** | **Length (Å)** | **Bond** | **Length (Å)** |
| Yb(1)-O(2) | 2.319(7) | Pd(4)-O(2) | 1.980(7) |
| Yb(1)-O(2)#1 | 2.319(7) | Pd(4)-O(2)#1 | 1.980(7) |
| Yb(1)-O(4) | 2.320(7) | Pd(4)-O(10)#1 | 2.010(8) |
| Yb(1)-O(4)#1 | 2.320(7) | Pd(4)-O(10) | 2.010(8) |
| Yb(1)-O(1)#1 | 2.320(7) | Pd(5)-O(2) | 1.996(7) |
| Yb(1)-O(1) | 2.320(7) | Pd(5)-O(4) | 2.000(7) |
| Yb(1)-O(3) | 2.326(8) | Pd(5)-O(11)#1 | 2.021(9) |
| Yb(1)-O(3)#1 | 2.326(8) | Pd(5)-O(12) | 2.056(7) |
| Pd(1)-O(1)#1 | 1.987(7) | Pd(6)-O(3) | 1.979(8) |
| Pd(1)-O(1) | 1.987(7) | Pd(6)-O(3)#1 | 1.979(8) |
| Pd(1)-O(5) | 1.999(7) | Pd(6)-O(13) | 2.028(8) |
| Pd(1)-O(5)#1 | 1.999(7) | Pd(6)-O(13)#1 | 2.028(8) |
| Pd(2)-O(2)#1 | 2.002(7) | Pd(7)-O(4) | 1.979(8) |
| Pd(2)-O(1) | 2.007(8) | Pd(7)-O(3)#1 | 1.995(7) |
| Pd(2)-O(6) | 2.020(9) | Pd(7)-O(15)#1 | 2.029(9) |
| Pd(2)-O(7) | 2.052(7) | Pd(7)-O(14) | 2.038(7) |
| Pd(3)-O(3) | 1.968(7) | Pd(8)-O(4)#1 | 1.986(7) |
| Pd(3)-O(1) | 1.977(8) | Pd(8)-O(4) | 1.986(7) |
| Pd(3)-O(8) | 2.010(7) | Pd(8)-O(16) | 2.006(7) |
| Pd(3)-O(9) | 2.024(8) | Pd(8)-O(16)#1 | 2.006(7) |
| Symmetry transformations used to generate equivalent atoms: #1: x,-y+3/2,z. | | | |

| **Lu-JUB-1** | | | |
| --- | --- | --- | --- |
| **Bond** | **Length (Å)** | **Bond** | **Length (Å)** |
| Lu(1)-O(3) | 2.312(6) | Pd(4)-O(10)#1 | 1.975(7) |
| Lu(1)-O(3)#1 | 2.312(6) | Pd(4)-O(10) | 1.975(7) |
| Lu(1)-O(2) | 2.312(6) | Pd(4)-O(2)#1 | 1.999(6) |
| Lu(1)-O(2)#1 | 2.312(6) | Pd(4)-O(2) | 1.999(6) |
| Lu(1)-O(1) | 2.314(6) | Pd(5)-O(2) | 1.978(6) |
| Lu(1)-O(1)#1 | 2.314(6) | Pd(5)-O(3) | 1.989(6) |
| Lu(1)-O(4) | 2.325(6) | Pd(5)-O(12) | 2.006(7) |
| Lu(1)-O(4)#1 | 2.325(6) | Pd(5)-O(11) | 2.032(6) |
| Pd(1)-O(1)#1 | 1.999(6) | Pd(6)-O(3) | 1.986(6) |
| Pd(1)-O(1) | 1.999(6) | Pd(6)-O(3)#1 | 1.986(6) |
| Pd(1)-O(5)#1 | 2.024(8) | Pd(6)-O(13) | 2.017(7) |
| Pd(1)-O(5) | 2.024(8) | Pd(6)-O(13)#1 | 2.017(7) |
| Pd(2)-O(1)#1 | 1.971(6) | Pd(7)-O(4) | 1.982(6) |
| Pd(2)-O(2) | 1.987(6) | Pd(7)-O(3) | 1.996(6) |
| Pd(2)-O(7) | 2.038(6) | Pd(7)-O(14) | 2.005(7) |
| Pd(2)-O(6) | 2.056(7) | Pd(7)-O(15) | 2.038(7) |
| Pd(3)-O(1)#1 | 1.969(6) | Pd(8)-O(4)#1 | 1.976(6) |
| Pd(3)-O(4) | 1.989(6) | Pd(8)-O(4) | 1.976(6) |
| Pd(3)-O(9) | 2.031(7) | Pd(8)-O(16) | 2.019(7) |
| Pd(3)-O(8) | 2.037(7) | Pd(8)-O(16)#1 | 2.019(7) |
| Symmetry transformations used to generate equivalent atoms: #1: x,-y+3/2,z | | | |


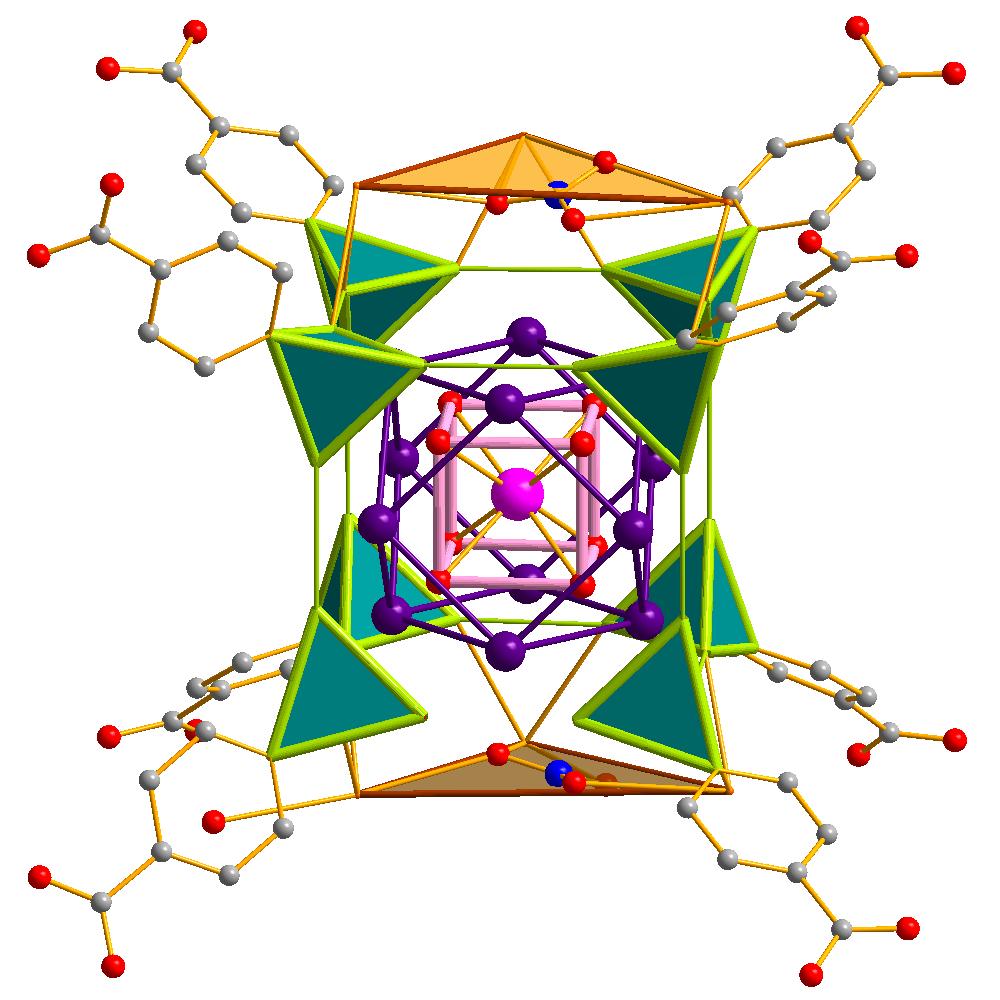

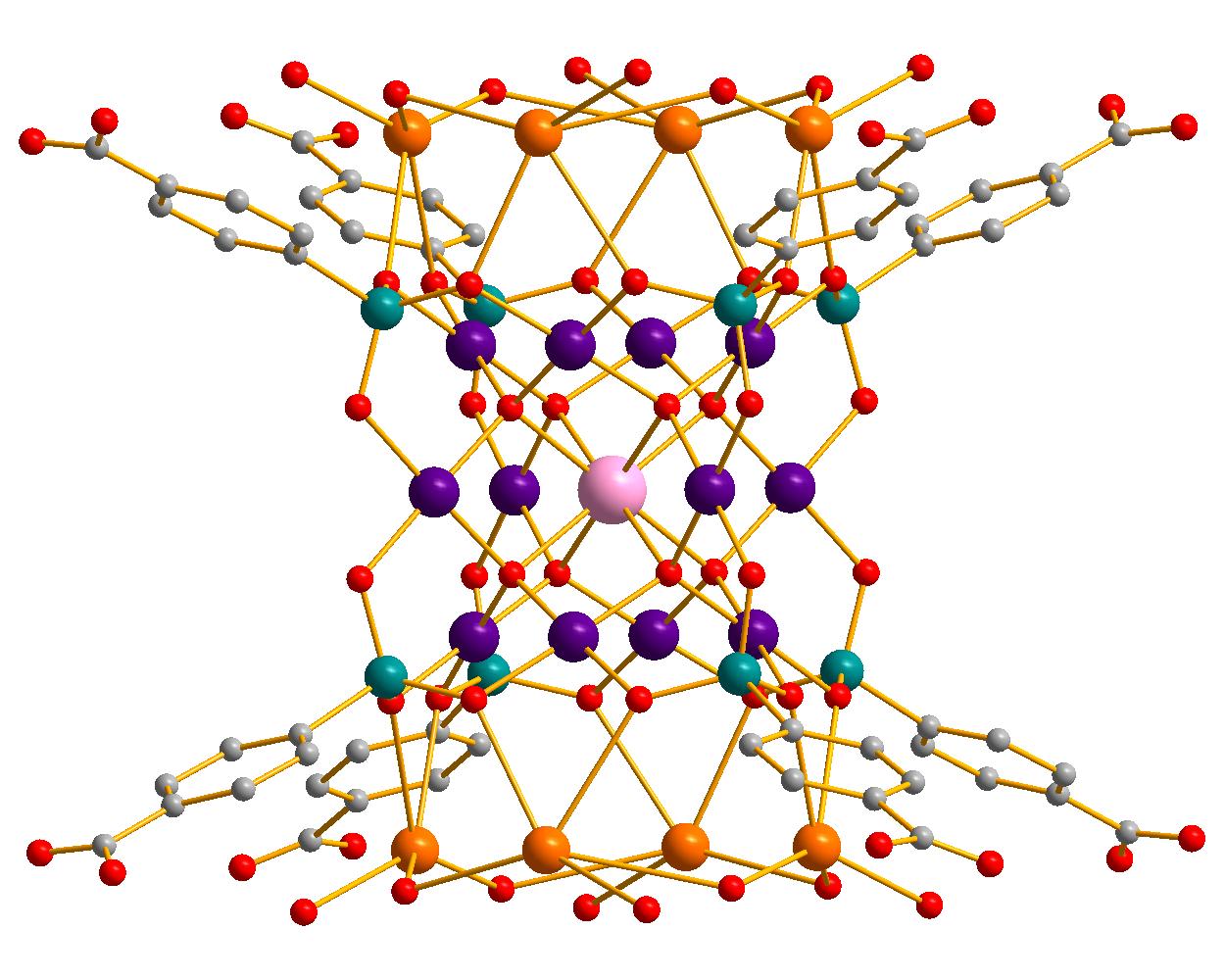
**Figure S1.** The [(Ce^III^)Pd_12_Ba_6_(μ_4_-O)_8_(CPA)_8_]^–^ or [(Th^IV^)Pd_12_Ba_6_(μ_4_-O)_8_(CPA)_8_] units. Colour code: Pd = purple balls, As = green balls, Ce/Th = pink ball, O = red balls, C = grey balls.

**Figure S2a.** The [{LnO_8_}{Pd}_12_{ArAs}_8_}] nano-cubic unit decorated on two of its sides by the fully occupied trinuclear Ba_3_ oxo-cluster. Colour code: Pd = purple balls, Arsonate = green polyhedra, Ln = dark pink ball, O = red balls, C = grey balls, N (of the bridging nitrate) = blue balls.


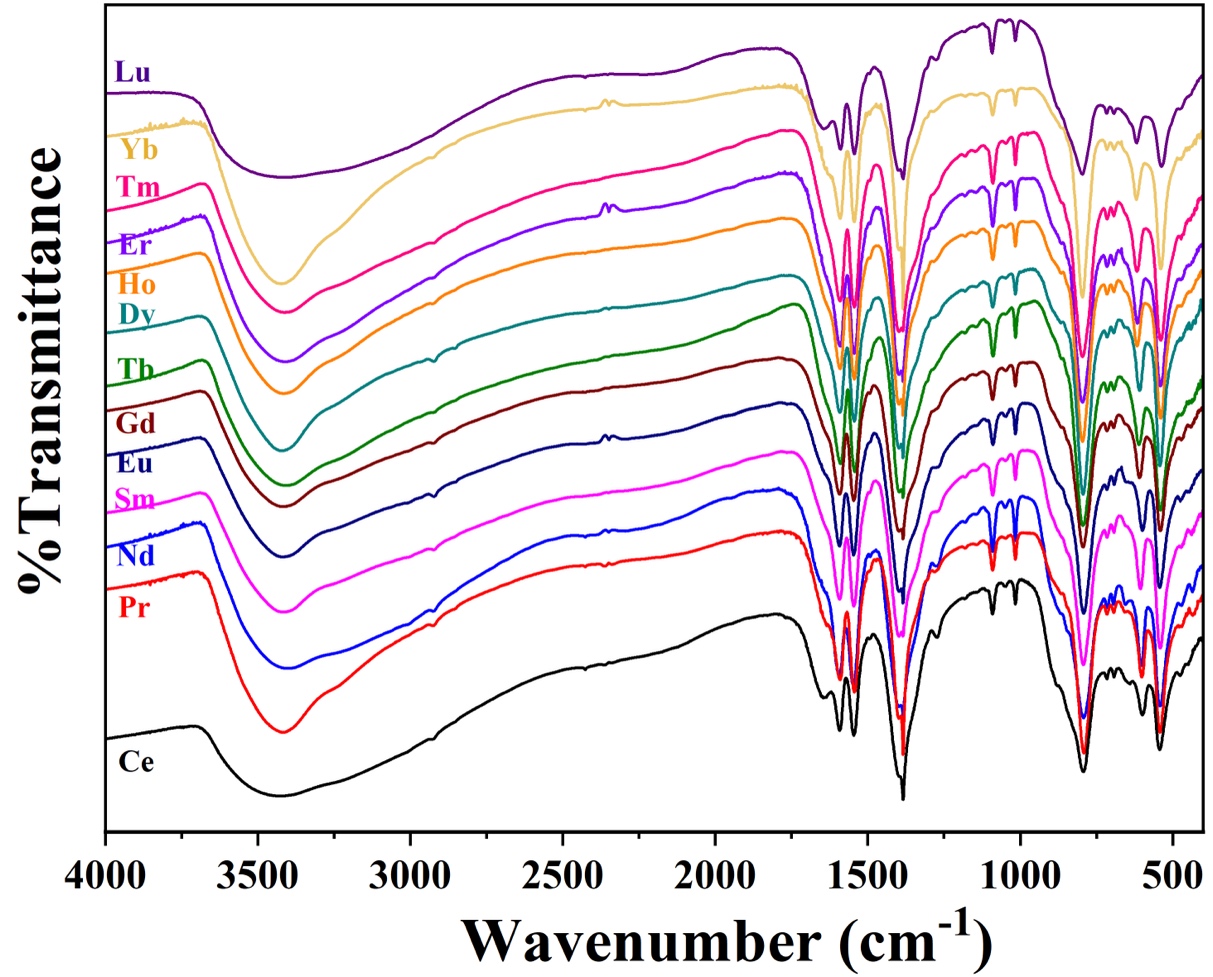

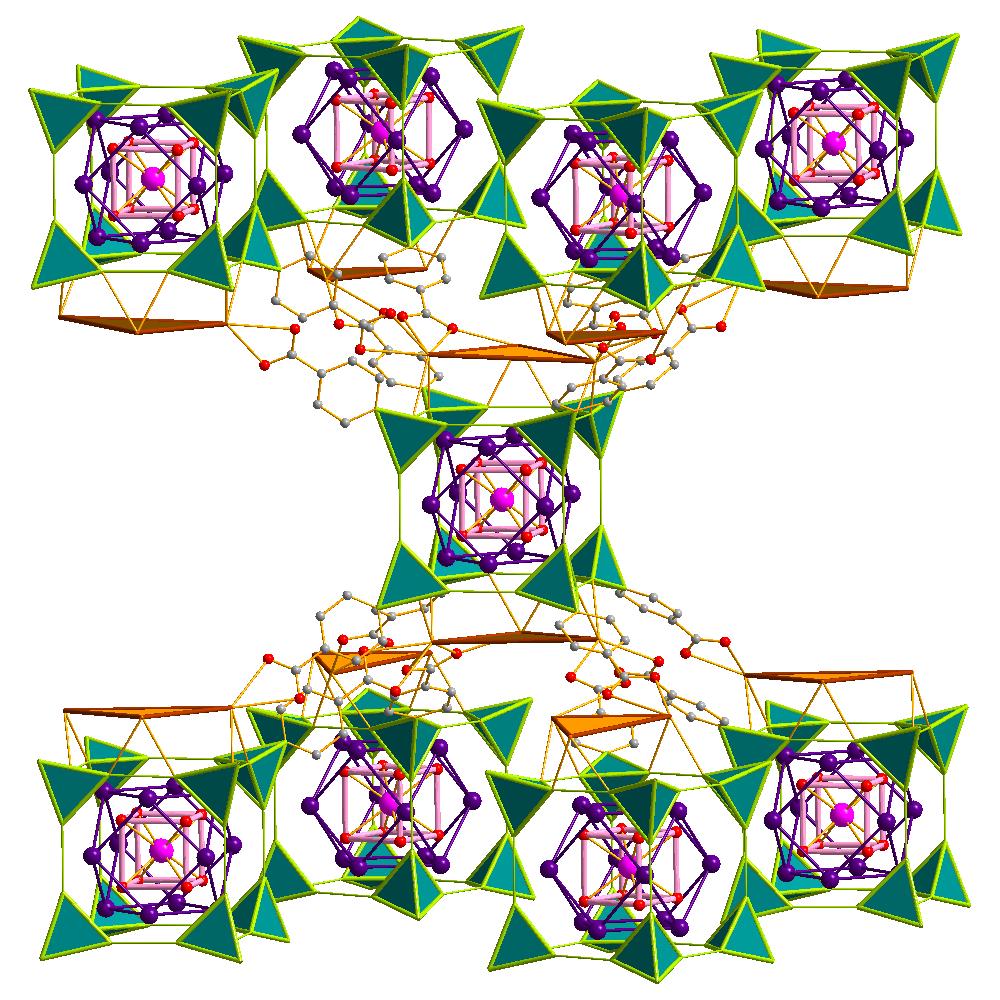
**Figure S2b.** The eight connected SBU in **Ln-JUB-1**.

**Figure S3.** Comparison of the IR spectra of **Ln-JUB-1** with **Ce-JUB-1**.

**Figure S4.** Comparison of the Thermogravimetric Analysis (TGA) curves of **Ce-JUB-1**, **Pr-JUB-1**, and **Lu-JUB-1**.
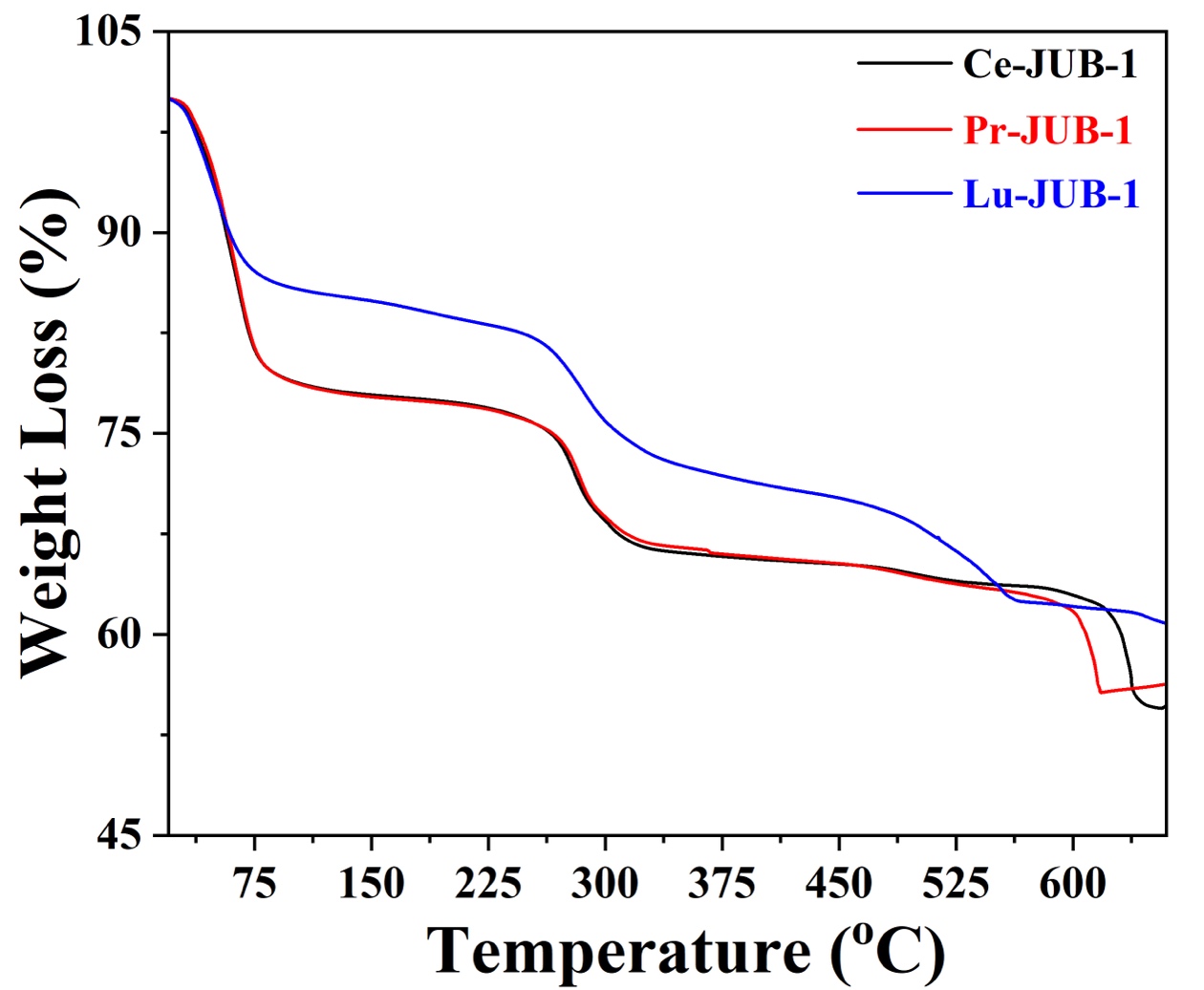

Supplement: Supplementary file 1 — Supporting Information [file ASIA-20-e00737-s002.docx]
